# Optimizing Web Links Used in Digital Vaccination Invitations to Raise Trust and Booking Intention: 3 Online Randomized Controlled Trials

**DOI:** 10.2196/85480

**Published:** 2026-08-25

**Authors:** Claire M Oakley, Hazel Sayer, Dawn Holford, Wändi Bruine de Bruin, Gaëlle Vallée-Tourangeau, Tim Chadborn, Miroslav Sirota, Marie Juanchich

**Affiliations:** 1 Department of Psychology, University of Essex, Wivenhoe Park, Colchester, CO4 3SQ, United Kingdom, 44 120 687 38 12; 2 School of Experimental Psychology, University of Bristol, Bristol, United Kingdom; 3 Schaeffer Institute for Public Policy and Government Service, University of Southern California, Los Angeles, CA, United States; 4 Department of Management, Kingston University, London, United Kingdom; 5 Public Health England, UK Health Security Agency, London, United Kingdom

**Keywords:** digital communication, health communication, weblinks, URL, trust, fluency, phishing, vaccination uptake

## Abstract

**Background:**

People are encouraged to respond swiftly to digital health invitations, but they can be (rightly) skeptical about their legitimacy.

**Objective:**

Drawing on digital communication theory and psychological science, we hypothesized that easy-to-read web links that facilitate participants’ ability to identify the health organization as the website host would improve trust in and user engagement with digital communications.

**Methods:**

In 3 double-blind, randomized online experiments, adult UK residents were recruited via the online platform Prolific (experiment 1: N_1_=569) or Qualtrics (experiment 2: N_2_=596; experiment 3: N_3_=1993). In each experiment, participants read a hypothetical email invitation for a COVID-19 vaccine from the UK’s National Health Service (NHS). Participants reported trust in the email (primary outcome), whether the web link was easy to read, who they thought the website host was, and their intention to book an appointment via the web link. We manipulated the booking web link (between-participants, double-blind allocation via the Qualtrics randomizer). Across experiments, the control group read the email containing a deactivated NHS vaccination booking web link: “accurx.thirdparty.nhs.uk/r/aafwaczmd5.” In experiment 1, participants read the email with the control link (randomized n=300, analyzed n=286) or an experimental clear link (“vaccine-booking.nhs.uk”; randomized n=301, analyzed n=283). In experiment 2, participants read the email with the control link (randomized n=203, analyzed n=201) or one of 2 experimental links: a shortened web link (“https://bit.ly/3GtTL0c”; randomized n=201, analyzed n=201) or a text-embedded link (“book here”; randomized n=196, analyzed n=194). In experiment 3, participants read the email with the control link (randomized n=655, analyzed n=655), the clear link (randomized n=669, analyzed n=668), or the text-embedded link (randomized n=670, analyzed n=670).

**Results:**

Across experiments, the control web link was poorly perceived, with most participants (729/1142, 63%) unsure or unlikely to use it. Relative to the control web link, the clear web link improved trust (β coefficient_Expe1_=0.26, 95% CI 0.18-0.36; β coefficient_Expe3_=0.24, 95% CI 0.19-0.30). The text-embedded web link also improved trust (β coefficient_Expe2_=0.18, 95% CI 0.09-0.27; β coefficient_Expe3_=0.17, 95% CI 0.11-0.22), but the shortened web link did not (β coefficient_Expe2_=0.01, 95% CI −0.09 to 0.11). Across experiments, improved host identification and ease of reading explained increased trust, which was significantly associated with increased booking intention. The clear and text-embedded web link (vs control) effects were robust when controlling for demographics.

**Conclusions:**

We extend digital communication research by investigating how web link design can reduce people’s justified suspicion in health messaging. Moving beyond the previous research focus on health message content, we experimentally test the role of web link wording. We bring causal evidence that easier-to-read links that have easy-to-identify host health institutions increased trust and intention to use the link. We provide simple, practical design guidance to improve real-world engagement with digital health communications.

## Introduction

### Background

Health care providers in the United Kingdom and elsewhere have expanded their communication channels, using social media and electronic messaging to improve patient engagement in preventive medicine and routine check-ups [1,2]. SMS text and email reminders have been cost-effective health interventions, increasing health check uptake [3], reducing missed appointments [4], and boosting vaccination rates [5,6]. The widespread adoption of digital communication thus has the potential to increase health care efficiency and quality of care [7]. The UK COVID-19 vaccination rollouts built on previous successes with digital invitations, with the National Health Service (NHS) sending millions of invitations to facilitate online booking of COVID-19 vaccine appointments [1]. However, the COVID-19 pandemic saw an explosion of cybercriminals impersonating reputable health organizations in phishing attempts (an attempt to steal private information) [8]. Recipients may therefore doubt the invitations’ authenticity. Genuine and nefarious messages both feature web links that lead to either a vaccination booking website or an attempt to steal personal information or money [9,10]. This research focuses on how the web links embedded within health invitation messages shape trust or distrust and booking intention.

The increase in COVID-19–related phishing attempts and the widespread media reporting of them may have undermined public trust [11]. Scammers impersonated health institutions in many countries, such as the United Kingdom [12], Australia [13], and France [14]. In 2022, 61% of all cyberfraud reported in England was related to COVID-19, with fraudsters mimicking NHS vaccination and testing invitations [15]. In 2020, Google reported that scammers were sending 18 million COVID-19–related hoax emails—including 10 million phishing attempts—every day [8]. These incidents potentially undermined public trust in using web links to book vaccine appointments and underscored the importance of adapting official digital health communication. Experimental work often focuses on health message *content* to increase trust and user engagement, such as how risk framing influences vaccination intention [16] or how to best present vaccine benefits [17,18]. Other research examines the characteristics of scam messages, such as the presence of typos [19], or pressure to meet a short deadline [20]. However, there is little research on the design of embedded web links in (health) messages and how their design may encourage or discourage recipients to click through.

According to information scent theory, web links emit an “information scent” that affects recipients’ perceptions of the linked website [21]. If the link accurately describes a webpage relevant to the recipients’ goals, they are more likely to click on it. It is, however, notoriously difficult to decide whether a link leads to a fraudulent or legitimate website [22]. Some technical features can be used to assess safety, including whether web links use specific protocols (eg, “https”) or official domains (eg, “.gov”) [19,23]. Beyond the web link’s technical features, we posit that their wording matters too. Moreover, 2 web link features could improve recipients’ trust perceptions and their intention to use the link. One way is to enhance the web link’s text to make it easier to identify the website’s host. Making the website host clearer to the reader echoes public advice to question whether the website host is known and trusted [24]. For example, public eHealth services are only trusted if the public can identify and already trust the service provider [25]. Vaccination booking links often include the name of a trusted health organization within the link, which is expected to elicit trust [26]. However, recipients might be unable to identify the organization if the presence of other words and nonsensical characters, as well as the absence of spaces between words, makes it difficult to parse the correct information.

The second web link feature follows from this notion of difficulty in parsing content. How easy or difficult it is to read a web link—a concept known as metacognitive fluency in cognitive psychology—might shape how much the link is trusted. According to fluency theory, reading ease shapes people’s perception beyond what is described literally [27]. For example, when analyzing real-world stock market data, people were more willing to buy stocks with fluent names like “Barnings” than those with hard-to-read names like “Ightsbry” [28]. Similarly, fictitious drugs or food additives with disfluent names, such as “Cytrigmcmium,” were perceived as more dangerous than those with more fluent names, such as “Fastinorbine” [29,30]. Fluency was also directly associated with trust perception in economics games, where people entrust more money to people with fluent names than to people with difficult-to-read names [31]. Text fluency is enhanced through the use of familiar words [31] that are easy to pronounce [28] and shorter [32]. Following fluency theory [27], we can expect that easy-to-read or shorter web links will feel more trustworthy and trigger greater user engagement than web links containing a long nonsensical string of letters and numbers. In practice, some web links used in vaccination invitation communications during the pandemic were *not* easy to read or short, and obscured the website host, such as the web link used by the NHS (“accurx.thirdparty.nhs.uk/r/aafwaczmd5”). Information scent theory and fluency theory together would posit that this would hinder recipients’ trust and willingness to use the link to book their appointment.

If it is not possible to amend the link to make it easier to read and to clarify the website host, “hiding” the link is an alternative option. The link can be hidden in descriptive anchor text, or by using a shortened URL service such as “bit.ly.” These methods could also improve fluency and host identification. Anchor text is the clickable text of a hyperlink, which can be tailored to be concise and easy to read. For example, by using a text-embedded web link: “Book your vaccine here.” Shortened URLs might merely improve fluency because they are shorter. A limitation of hiding links (in text or shortened URL) is that it is a classic phishing technique to obscure the (real) website host [33]. Recipients must therefore rely on other information to verify authenticity, such as the sender’s information from the message or email content.

### Objectives

We conducted 3 experiments using hypothetical but realistic digital vaccination booking invitations to examine how manipulating the web link impacted recipients’ trust in the email (the primary outcome) and their intentions to book a vaccination appointment using the link. The invitations were modeled on NHS digital COVID-19 communications. Across experiments, the control condition featured a deactivated NHS COVID-19 vaccination web link (“https://accurx.thirdparty.nhs.uk/r/aafwaczmd5”). Experiment 1 compared this control link with an improved, clear web link (“https://vaccine-booking.nhs.uk”), hypothesizing improved performance (hypothesis A). The clear web link was expected to facilitate host identification (hypothesis A_1_) and to be easier to read (hypothesis A_2_), thereby increasing trust (hypothesis A_3_) and booking intention (hypothesis A_4_). Experiment 2 tested whether concealing the web link in text (text-embedded: “Book your vaccine here.”) or in a shortened URL (“https://bit.ly/3GtTLoc”) would improve performance (hypothesis B). We hypothesized that the 2 concealed links would facilitate host identification (hypothesis B_1_) and ease of reading (hypothesis B_2_), thereby increasing trust (hypothesis B_3_) and booking intention (hypothesis B_4_). Experiment 3 tested the control, clear, and text-embedded web links in a more ethnically diverse sample (hypotheses A_1_-A_4_ and B_1_-B_4_).

## Experiment 1

### Methods

#### Open Science

Experiment 1, and the following experiments, were preregistered on the AsPredicted website before data collection (experiment 1: #84106, “https://aspredicted.org/HR5_864,” December 29, 2021; experiment 2: #84816, “https://aspredicted.org/STF_WNP,” January 11, 2022; and experiment 3: #86763, “https://aspredicted.org/P7B_JBM,” February 1, 2022). All experiments proceeded as described in the preregistrations.

AsPredicted preregistration was deemed sufficient to reduce the risk of bias [34,35] as those preregistrations include hypotheses, experimental conditions, measures, sample size target, and statistical plans. To enhance transparency, the studies were registered retrospectively on a World Health Organization (WHO)–recognized trial registry, that is, ClinicalTrials.gov (experiment 1: NCT07516600, March 31, 2026; experiment 2: NCT07532967, March 31, 2026; experiment 3: NCT07538349, April 15, 2026). The AsPredicted preregistration protocols, ClinicalTrials.gov registration, materials, and data are available on the Open Science Framework (OSF) website [36].

Each of the 3 studies was conducted in the United Kingdom, using the NHS as the email sender, and in the United States, using a fictitious pharmacy as the sender. Only the results for the United Kingdom study version are reported here. The results using the fictitious pharmacy are reported in Supplementary Materials on the OSF website [36].

#### Ethical Considerations

##### Ethics Approval

All 3 experiments received ethical approval from the University of Essex Ethics Committee (ETH 2122‐0265). Informed consent was obtained from all participants before participation, and individuals who did not provide consent could not proceed with completing the study. The study was introduced as focusing on COVID-19 vaccination. Potential participants were informed that the study had obtained ethics approval. Potential participants were informed about risks and benefits involved before deciding to take part. Participants were paid via the panel provider (Prolific for experiment 1, Qualtrics for experiments 2 and 3). In each study, participants could only participate and validate their participation once. All responses were anonymous. We did not gather information that could be used to identify individuals; therefore, individual participants cannot be identified from the information reported in the main text or multimedia appendix. Study information and consent questions are provided with the full survey details through the OSF [36].

##### Participants and Patients Involvement

The study materials were reviewed by members of a “Participants and Patients Involvement” panel. These respondents were paid £50 (approximately US $67.8) each to share in-depth feedback on study information clarity, the wording of questions, and response options that led to the current study version.

##### Harms

The ethics panel agreed that there were no expected or unexpected harms in the experiments. No adverse events, unintended effects, or participant complaints were reported in any experiment.

### Participants

Using quota sampling recruitment, we invited 600 adult UK respondents from the Prolific verified online participants pool to complete the study, aiming for a 50% gender goal. Participants received £2.50 (approximately US $3.39) for taking part (median completion time 15.5, IQR 12.3-20.5 min; equivalent to £9.68/h [approximately US $13.13]). We received 614 responses. Of this initial sample, 13 did not include any data for this study (3 did not give consent to proceed and 10 did not finish the experiment). We excluded 32 cases where participants failed at least one of 2 preregistered attention check questions, designed to confirm that participants were reading the questions. A minimum survey completion time of 5 minutes was set to filter out speeders. The resulting sample size of 569 participants was sufficient to detect a small to medium effect of the link manipulation relative to the control on trust perception in an analysis of variance, *f*=0.15 (assuming α=.05, power=0.95).

Participants’ ages ranged from 18 to 88 (mean 41.25, SD 14.61) years. Our sample comprised 49% (276/569) women, 50% men (283/569), and 1% (8/569) nonbinary, with an ethnic composition of 86% (486/569) White, 7% (40/569) Asian, 4% (22/569) Black, 3% (15/569) Mixed or Multiple ethnicities, and 1% (6/569) Other. The study took place in the United Kingdom in January 2022, when the first COVID-19 vaccine had become available to all adults. The booster vaccine was also available to adults who had received their first COVID-19 vaccination over 3 months before [37]. Most (507/569, 89%) of the participants had received their first COVID-19 vaccination, and 72% (359/569) of those who were eligible had received their booster vaccination. Refer to Multimedia Appendix 1 for a more detailed sample description.

### Trial Design

This online study used a between-participants experimental design, in which participants were randomly assigned to one of 2 parallel conditions (1:1 allocation)—a control or an experimental condition. Both groups read a COVID-19 vaccination invitation email, but the web link used in the email differed. The control group read a vaccination invitation email with the web link that was used by the NHS in SMS text message vaccination invitations in 2021 and 2022 (“accurx.thirdparty.nhs.uk/r/aafwaczmd5”). The experimental group read the same email; the web link was a “clear” web link that was easy to read and had an easily identifiable website host (“https://vaccine-booking.nhs.uk”). Based on the random allocation of the 569 participants via the automatic Qualtrics randomizer, 286 participants were allocated to the control condition, and 283 participants to the experimental condition. Researchers were blind to the allocation of participants to each condition.

### Materials and Procedure

Data collection took place in the United Kingdom in early January 2022 and ceased once the recruitment target was met. The study participants were recruited from the Prolific platform’s verified participant pool and were directed to the study hosted on the University of Essex Qualtrics online platform. The only eligibility criteria were to be older than 18 years of age and a UK resident. The study took place during the COVID-19 pandemic, when the Omicron variant—a more infectious but less severe COVID-19 variant—caused a substantial increase in COVID-19 infections [38]. Despite a decrease in severity, the size of the epidemic wave caused an increase in deaths and hospitalizations [38].

Participants accessed the study individually via a web browser on their own devices as part of an online survey. After obtaining informed consent at the beginning of the survey, participants were asked if they had received their first dose of the COVID-19 vaccine. The study started with questions about vaccination attitudes for a separate study (eg, vaccine risk perception, vaccine effectiveness, and subjective social norm). Participants were then presented with the materials for this study.

**Figure 1.**
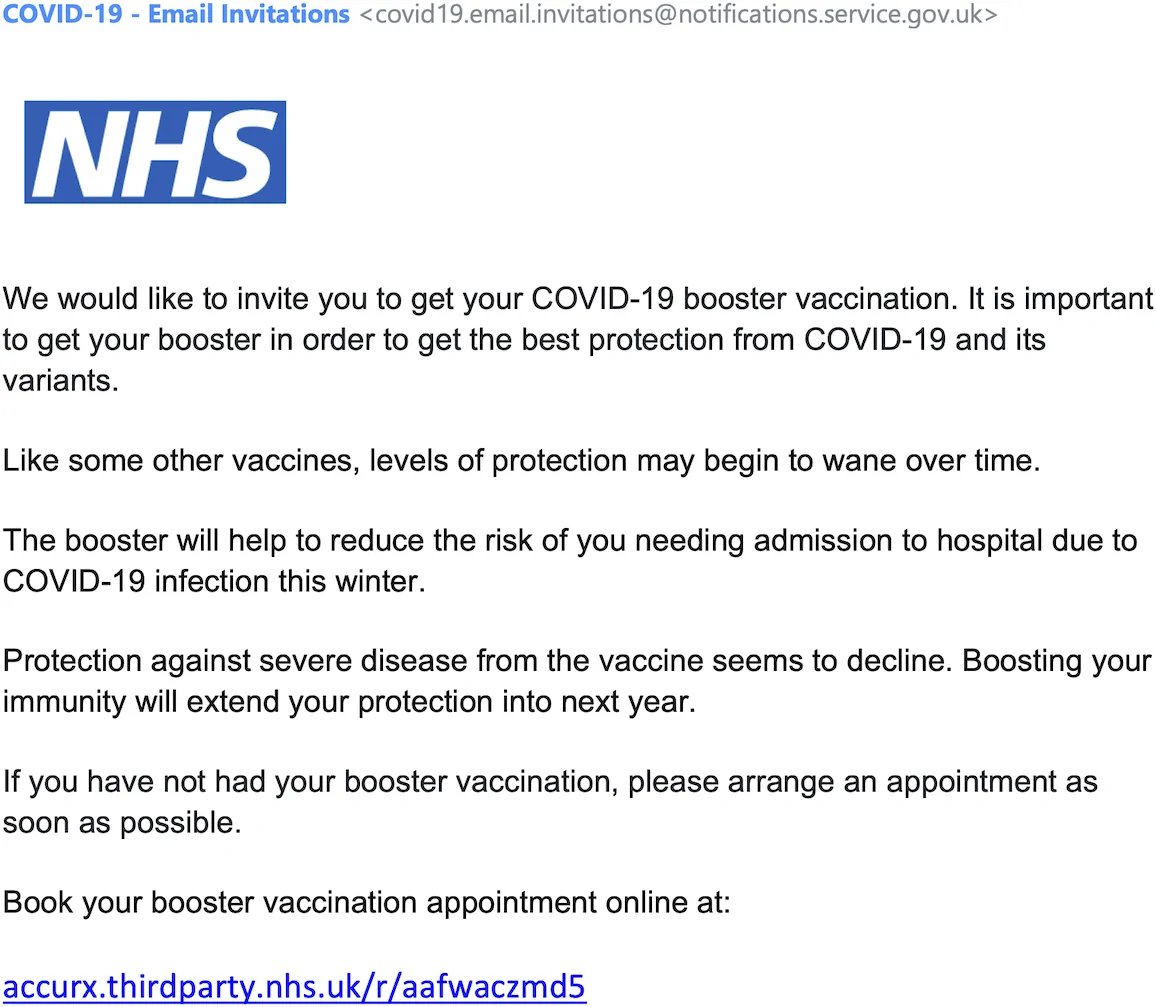
Email invitation used in experiments 1‐3, with the control web link used by the National Health Service (NHS) in the United Kingdom for 2021‐2022 COVID-19 vaccination invitations.

Participants read a hypothetical vaccination invitation email as illustrated in Figure 1. The email contained either the control or the experimental “clear” web link. Random assignment was implemented using Qualtrics’ built-in randomizer, concealing allocation and ensuring double-blind allocation of participants to conditions. As the invitations were hypothetical, we did not use active links, so participants were unable to click on them in the email. Immediately after reading the hypothetical email, participants rated the degree to which they thought the email appeared to be suspicious or trustworthy (the primary outcome) on a 5-point Likert scale (1=very suspicious to 5=very trustworthy), how likely they were to click on the web link to book an appointment (1=very unlikely to 5=very likely), and evaluated how easy to read the web link was (a measure of fluency; 1=very difficult to 5=very easy). Participants then answered who they thought was hosting the web link, with four responses: “The NHS,” “Pharmacy US,” “A third party,” or “Not sure,” which were recoded as correct (NHS=1) or incorrect (any other answer=0). Finally, participants completed sociodemographic questions, indicating their age, ethnicity, gender, and education level.

### Statistical Methods

We report path analyses showing the effect of the link on trust perception and booking intention as well as the mediated effect of the link via correct host identification and perceived ease of reading of the web link. Further regression analyses tested the robustness of effects, controlling for gender, age, education, and ethnic minority status and, in experiment 3, their interactions. The preregistered analyses of variance testing the effect of the web link manipulation on trust, booking intention, and ease of reading, and the chi-square testing the web link manipulation on host organization identification show the same results and are reported in Multimedia Appendix 1. The distributions of the variables and the zero-order correlations between variables are available in Multimedia Appendix 1. There were no missing data in the experiments. In each experiment, data transformation, variance analyses, and chi-square tests were performed using IBM SPSS (version 28). Path analyses were implemented using the *lavaan* package (v. 0.6‐11) with RStudio (version 2022.01.07; Posit) and R (version 4.0.4; R Core Team).

### Results

**Figure 2.**
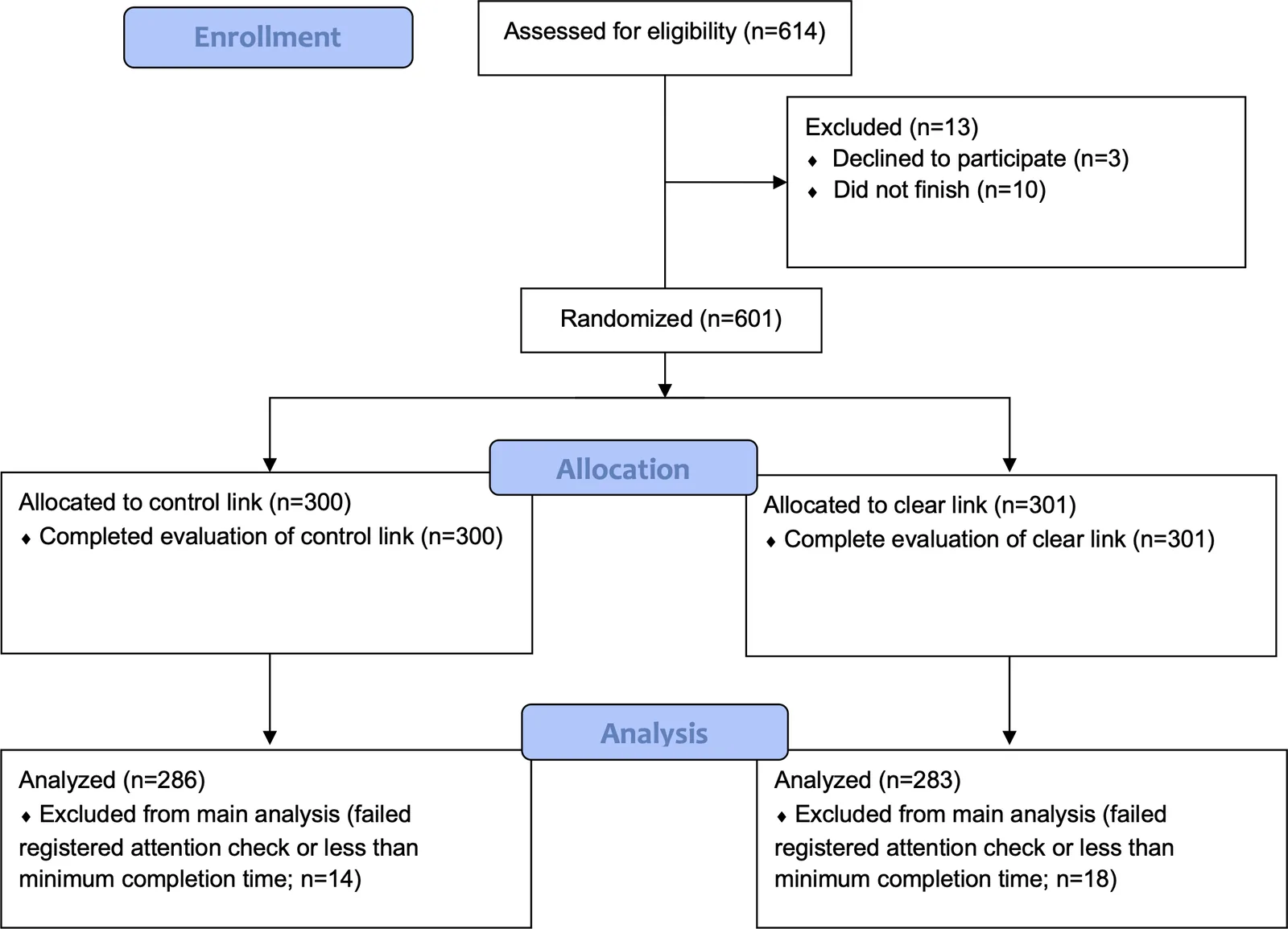
Participant flow chart for experiment 1. Exclusion decisions followed preregistered criteria: <5 minutes completion time or incorrectly answered 2 attention checks.

**Figure 3.**
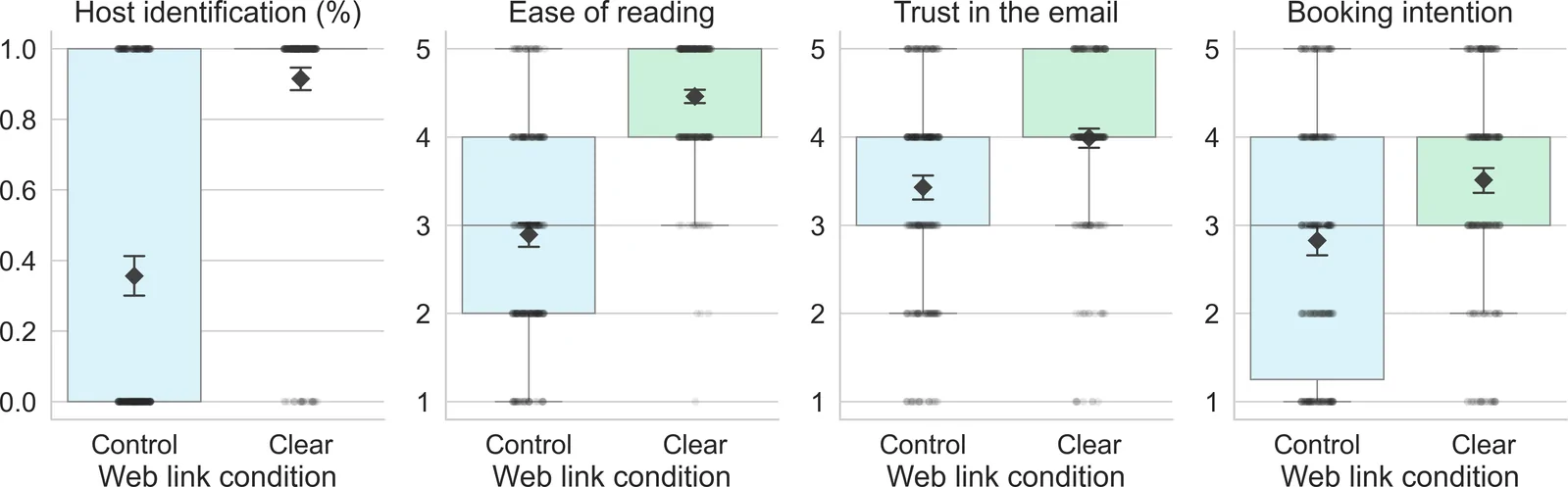
Relative to the control web link (in blue), the clear web link (in green) helped participants recognize the website host correctly, was judged easier to read, and the email in which the web link appeared was found more trustworthy. Participants reported greater intention to book their vaccination appointment via the web link (experiment 1: N=569; n=276, 49% female; n=486, 86% White British or Other; early January 2022). The means are shown in black diamonds with error bars showing with 95% CIs. The box represents the IQRs, and the error bars around the box the minimal and maximal values without outliers. The light gray circles show data points with slight horizontal jitter, so that wider and denser clusters represent more frequent answers. The host identification panel (leftmost) shows the proportion of correct interpretations (0: 0% and 1: 100%). The control link was the National Health Service (NHS) original vaccination invitation link “accurx.thirdparty.nhs.uk/r/aafwaczmd5” and the clear link was “https://vaccine-booking.nhs.uk.”

**Figure 4.**
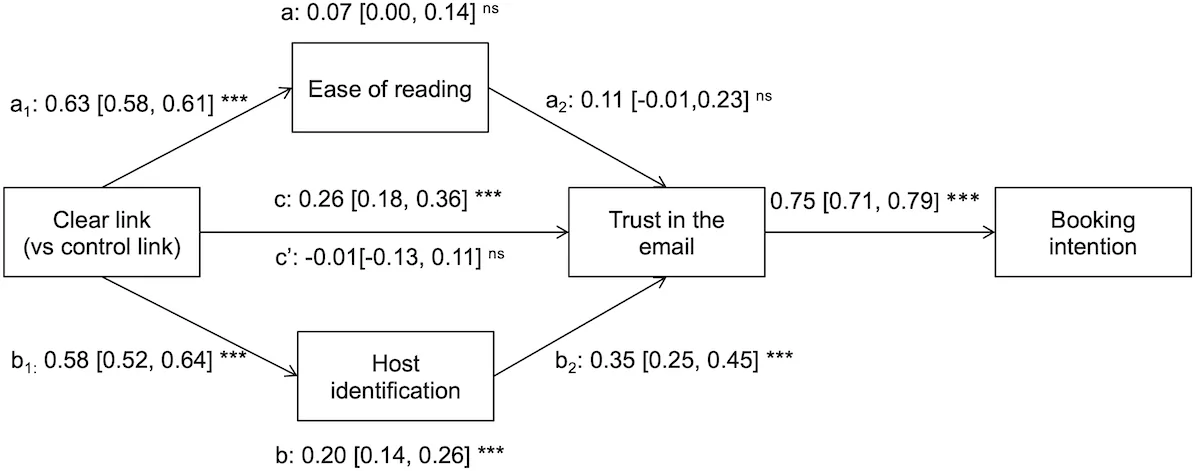
Effect of the clear web link (vs control) on correct identification of the host, ease of reading, trust in the email, and booking intention. The effect of the web link on trust was explained by improved correct host identification. The model shows β regression coefficients (experiment 1: N=569; n=276, 49% female; n=486, 86% White British or Other; early January 2022). Indirect effects of the link: a: via perceived ease of reading of the link, b: via correct host identification. Values are standardized, path analysis β coefficients with bias-corrected bootstrap 95% CIs from 5000 samples. ns: not significant. *** *P*<.001.

Most participants (184/286, 64%) who received the email containing the control link were unable to correctly identify the NHS as the web link’s host and judged the web link as slightly hard to read (refer to Figure 2 for the CONSORT [Consolidated Standards of Reporting Trials] participant flow chart [39]). As hypothesized, the clear web link resulted in improved host identification compared with the control link (259/283, 92% correct vs 102/286, 36%), was judged easier to read, led to greater perceived trust in the email, and fostered higher booking intentions (Figure 3). Of those participants who received the email with the control web link, 43% (123/286) of the respondents were skeptical about the trustworthiness of the email (responding either very suspicious, quite suspicious, or not sure), and 63% (181/286) were not keen to use the control link (responding either very unlikely, unlikely, or unsure). The path analysis (Figure 4) revealed that the positive effect of the clear web link (compared with the control link) on trust and booking intentions was partly explained by the improved identification of the website host (refer to β coefficient of the indirect effect, path b). Ease of reading (indirect effect, path a) did not explain the effect of the link on trust. The path between ease of reading and trust was not statistically significant, indicating that perceived ease of reading and host identification competed when predicting trust perception in the email. The analyses conducted on the full sample (without the preregistered data exclusions) also showed no significant mediation effect of ease of reading. The path model is reported in Multimedia Appendix 1. The clear web link’s positive effect on trust, compared with the control link, was robust after adding covariates of gender, age, education, and ethnic minority status in a regression analysis (Multimedia Appendix 1).

Experiment 1 shows how minor changes to a web link embedded in an email can change people’s perception of that email. The results show that using a web link that facilitates host identification and is easy to read increases trust and booking intention.

## Experiment 2

### Methods

#### Overview

In experiment 1, we found that improving the web link improved trust in the vaccination invitation email and booking intention, relative to a control link previously used nationally by the NHS. However, it is only sometimes possible or practical to change the website’s web link. In this follow-up experiment, we examined an alternative strategy that sidesteps the need to modify the link: concealing it—a strategy used by UK General Practitioner practices (based on the UK authors’ experience). We compared the control web link with a text-embedded web link (the “book here” web link) and a shortened web link using URL shortener services (“https://bit.ly/3GtTL0c”). In effect, this procedure did completely conceal the host from the URL, but the host could still be identified via other cues in the message (eg, message sender).

We expected that compared with the control web link, concealing the web link would make the web link easier to read and would facilitate host identification (hypotheses B_1_-B_2_) and therefore increase trust in the message and booking intention (hypotheses B_3_-B_4_).

#### Participants

We used Qualtrics as a panel provider for recruitment, setting quotas for gender, age, and ethnicity to match the UK population. Participants were paid by the panel provider directly. Of the 600 participants who took part, we excluded four cases who sped through the study (<5 min completion time) or did not correctly answer the 2 attention check questions included in the study. The 596 participants were all UK residents, 18 to 88 years of age (mean 44.74, SD 16.67 y). The sample comprised 51% (301/596) women, 49% (292/596) men, and 1% (3/596) nonbinary, with an ethnic composition of 87% (514/596) White British or Other, 7% (44/596) Asian, 3% (19/596) Black, 2% (13/596) Mixed or Multiple ethnicities, and 1% (6/596) Other. The sample size of 596 participants was sufficient to detect a small to medium effect when comparing the 3 web links regarding trust perception in a variance analysis, *f*=0.17 (assuming α=.05, power=0.95). Refer to Multimedia Appendix 1, for a more detailed sample description.

#### Trial Design

Participants were randomly assigned by an automatic Qualtrics randomizer to one of 3 parallel conditions (1:1:1 allocation): viewing a hypothetical COVID-19 vaccine invitation featuring either the control link (Figure 1) or one of 2 concealed link conditions: a text-embedded web link (the “book here” web link) or the shortened web link created using bit.ly services (“https://bit.ly/3GtTL0c”). Automated randomization through Qualtrics concealed allocation and ensured double-blind allocation to conditions. Based on the random allocation, of the 596 participants in this online study, 201 were allocated to the control condition, 194 to the text-embedded web link condition and 201 to the shortened web link condition.

#### Materials and Procedure

This study followed the same procedure as experiment 1, with the materials only altered to reflect the 2 new experimental web links being tested. Data collection took place from mid to late January 2022 and ceased once the recruitment target was met.

### Results

**Figure 5.**
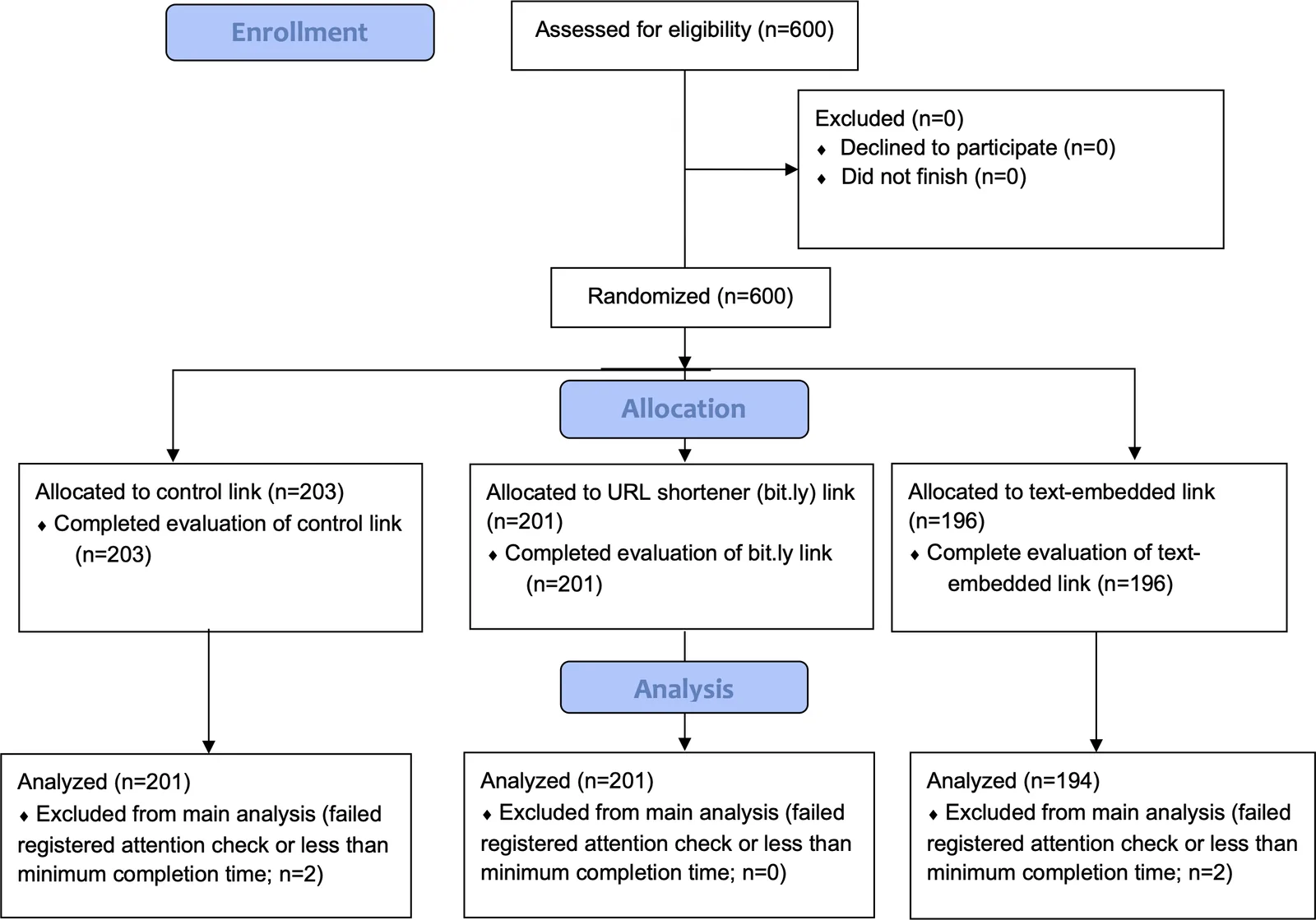
Participant flow chart for experiment 2. Exclusion decisions followed preregistered criteria: <5 minutes completion time or incorrectly answered 2 attention checks.

**Figure 6.**
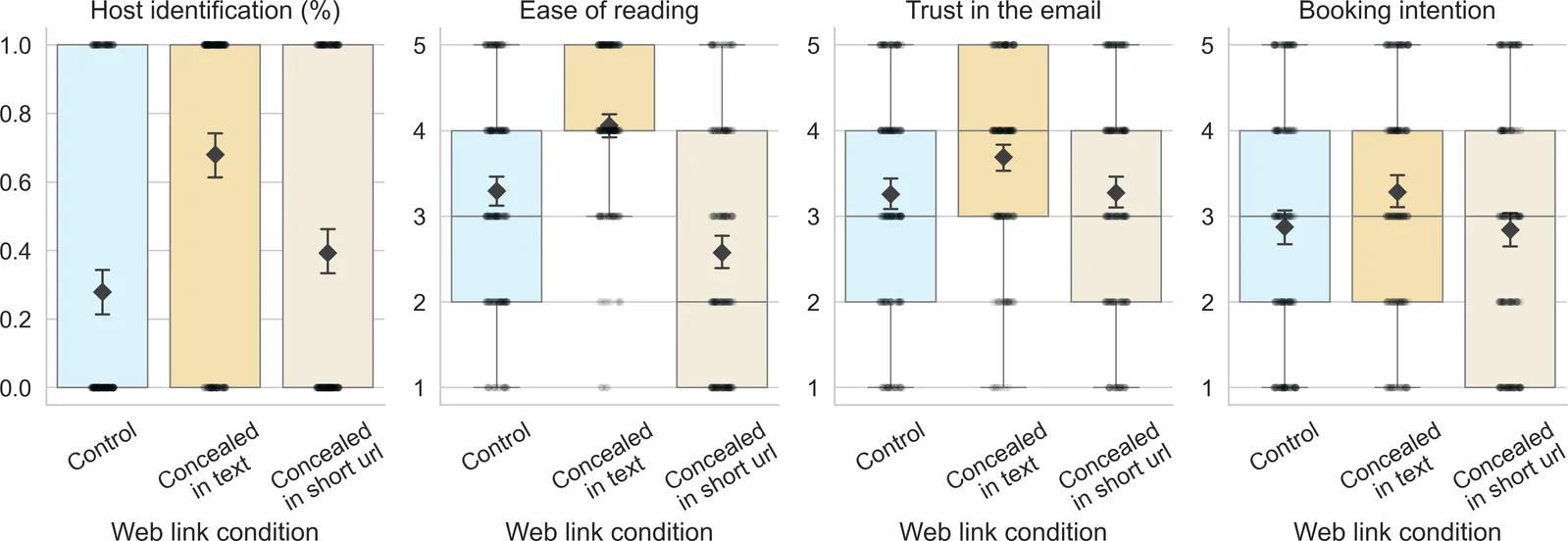
Relative to the control web link (in blue), concealing the web link in text (in orange) or in a shortened web link (in beige) helped participants recognize the website host correctly. Only the text-embedded web link was considered easier to read and had an overall positive effect on email trust and booking intention (experiment 2: N=596; UK population representative sample, mid-late January 2022). The means are shown in black diamonds with error bars showing 95% CIs. The box represents the IQR, the error bars show the minimal and maximal values without outliers. The light gray circles show data points with slight horizontal jitter, so that wider and denser clusters represent more frequent answers. The host identification panel (leftmost) shows the proportion of correct interpretations (0: 0% and 1: 100%). The control link was the National Health Service (NHS) original vaccination invitation link “accurx.thirdparty.nhs.uk/r/aafwaczmd5,” the 2 concealed links were the text-embedded web link “book here,” and the shortened link “https://bit.ly/3GtTL0c.”

**Figure 7.**
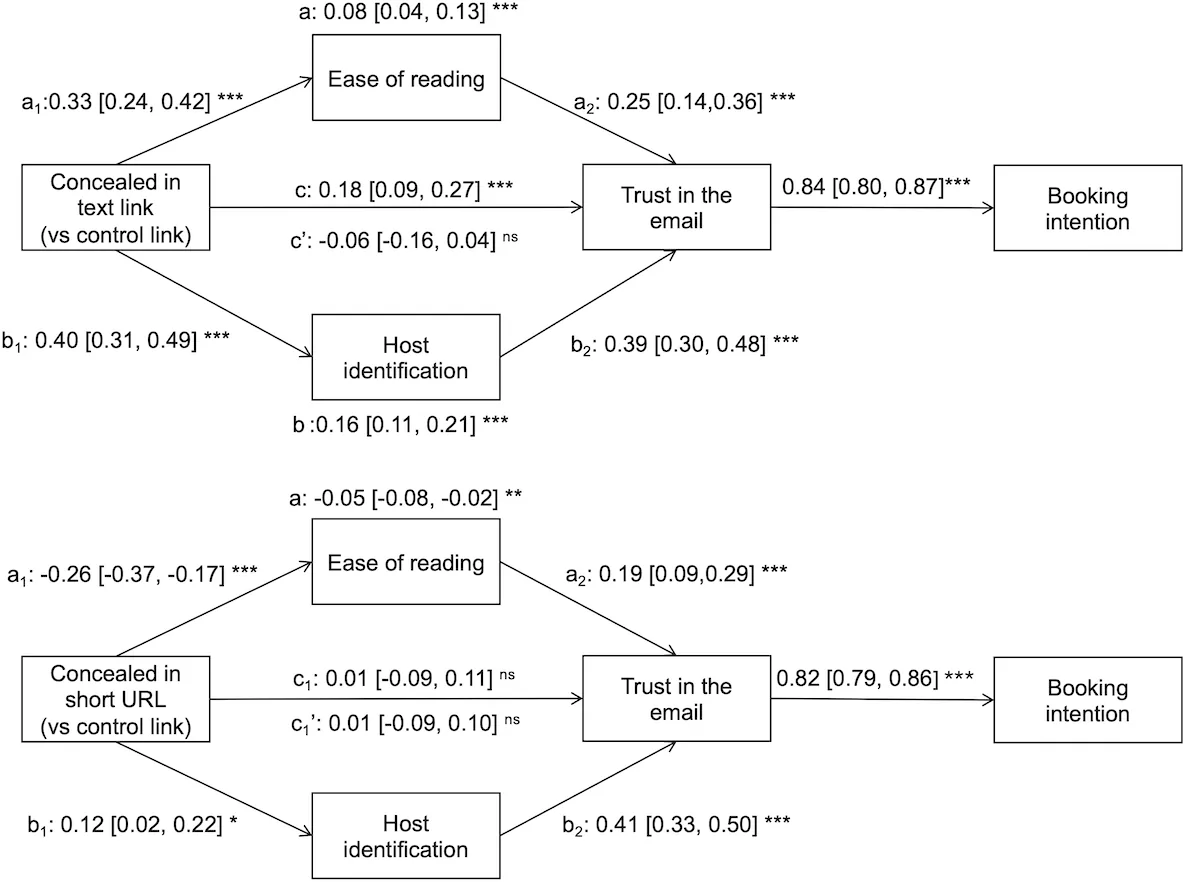
The text-embedded web link and the bit.ly link had an effect on trust and booking intention (vs control link) via the correct identification of the host and ease of reading. The models show β regression coefficients (experiment 2: N=596; UK population representative sample, mid-late January 2022). Indirect effects of the link: a: via ease of reading of the link, b: via correct host identification. Values are standardized, path analysis β coefficients, with bias-corrected bootstrap 95% CIs from 5000 samples. ns: not significant. *P<.05, **P<.01, ***P<.001.

Experiment 2 (refer to Figure 5 for the CONSORT participant flow chart [39]) replicated the finding of experiment 1, that the control link was perceived more negatively, resulting in lower website host identification, ease of reading, trust, and booking intention (Figure 6). Compared with the control web link, when the email included the text-embedded web link, participants were better able to identify the website host (132/194, 68% correct vs 56/201, 28%), the link was also perceived to be easier to read, and participants reported greater trust in the email and booking intention. The shortened web link also led to an increased identification of the website host compared to the control link (79/201, 39% correct vs 56/201, 28%), but the shortened link was perceived to be *less* fluent and did not yield a greater level of trust and booking intention (Figure 7). The path analysis effects are reported in Figure 7. We report the results of the mediation results here and those of the preregistered analyses (showing consistent findings) in Multimedia Appendix 1.

The path model shown in the upper panel of Figure 7 indicates that the effect of the text-embedded web link on trust was statistically explained by both the heightened correct host identification and reading ease. The model shown in the lower panel indicates that the bit.ly web link had the expected positive effect on trust via improved host identification. However, this effect was canceled out by the *negative* effect that the link had on trust because it was not perceived as easier to read. Therefore, the bit.ly web link did not improve trust perception or booking intention compared to the control web link.

The effect of the web link on trust was similar after adding gender, age, education, and ethnic minority status as covariates in a regression analysis (Multimedia Appendix 1).

Experiment 2 evidenced that concealing a web link in a vaccination invitation email can be a good strategy to improve trust, but the effectiveness depends on how the link is hidden. Our results show that although both the shortened bit.ly and the text-embedded links omitted the source in the URL, this did not prevent participants from identifying the NHS as the host. Most participants used visual cues, such as the NHS logo in the email letterhead, to determine the website host. However, the 2 concealed web links differed in perceived ease of reading. The shortened bit.ly web link was perceived as less easy to read and overall did not improve trust and booking intention. In contrast, concealing the web link by embedding it within a word was perceived as easier to read (and facilitated host identification), leading to higher levels of trust in the overall email and booking intention. The results emphasize the additive role of host identification and ease of reading.

## Experiment 3

### Methods

#### Overview

Experiments 1 and 2 show that a clear web link and a concealed, text-embedded web link are effective strategies that facilitate host identification, improve the perceived ease of reading the link, foster trust in the email, and increase booking intention. In experiment 3, we compare the benefits of improving the web link’s perceived ease of reading and including a clear host with concealing the web link by embedding it in text, relative to the control link in a larger and more ethnically diverse sample. In the United Kingdom, people from ethnic minority groups tend to trust health institutions less [40], which might undermine the benefits of recognizing a well-known health institution. This lower level of institutional trust is one of the factors that explains why vaccination uptake is lower among ethnic minority communities in the United Kingdom [41-43] despite these groups being more likely than the white majority to experience severe illness and death from COVID-19 [44].

#### Participants

The study was carried out in February-March 2022 via the Qualtrics survey platform. We used Qualtrics as a panel provider to recruit participants in the United Kingdom. We used quota samples on age and gender to reach UK-representative proportions but adjusted ethnicity quota to sample more participants from ethnic minority groups (up to 50%). Participants were paid by the panel provider directly. Of the 1994 UK respondents who passed the attention check, 1 was excluded for completing the study too quickly (a preregistered minimum time to complete the study of 5 min). The sample size of 1993 participants was sufficient to detect a small effect of the link manipulation relative to the control condition on trust perception in a variance analysis where 3 groups were compared, *f*=0.09 (assuming α=.05, power=0.95). Participants’ ages ranged from 18 to 89 (mean 45.63, SD 16.62) years. The sample comprised 51% (1009/1993) women, 49% (981/1993) men, <0.1% (2/1993) nonbinary, and <0.1% (1/1993) other gender identification, with an ethnic composition of 53% (1057/1993) White, 23% (470/1993) Asian, 13% (264/1993) Black, 9% (176/1993) Mixed or Multiple ethnicity, and 2% (51/1993) Other. Refer to Multimedia Appendix 1 for a more detailed sample description.

#### Trial Design

Using a between-participants design, participants in this online study were randomly allocated to one of 3 parallel conditions using the automatic Qualtrics randomizer (1:1:1 allocation): participants viewed a hypothetical COVID-19 vaccination invitation email using either the control link (“accurx.thirdparty.nhs.uk/r/aafwaczmd5”), the clear web link (“https://vaccine-booking.nhs.uk”), or the text-embedded web link (“book here”). Automated randomization through Qualtrics concealed allocation and ensured double-blind allocation to conditions. Based on the random allocation of the 1993 participants, 655 participants were allocated to the control condition, 668 participants to the clear link, and 670 participants to the text-embedded link condition.

#### Materials and Procedure

This study followed the same procedure as experiments 1 and 2, reusing the materials from experiment 1 for the control web link and clear web link condition, and the email containing the text-embedded web link from experiment 2. Data collection took place during February-March 2022 and ceased once the recruitment target was met.

### Results

**Figure 8.**
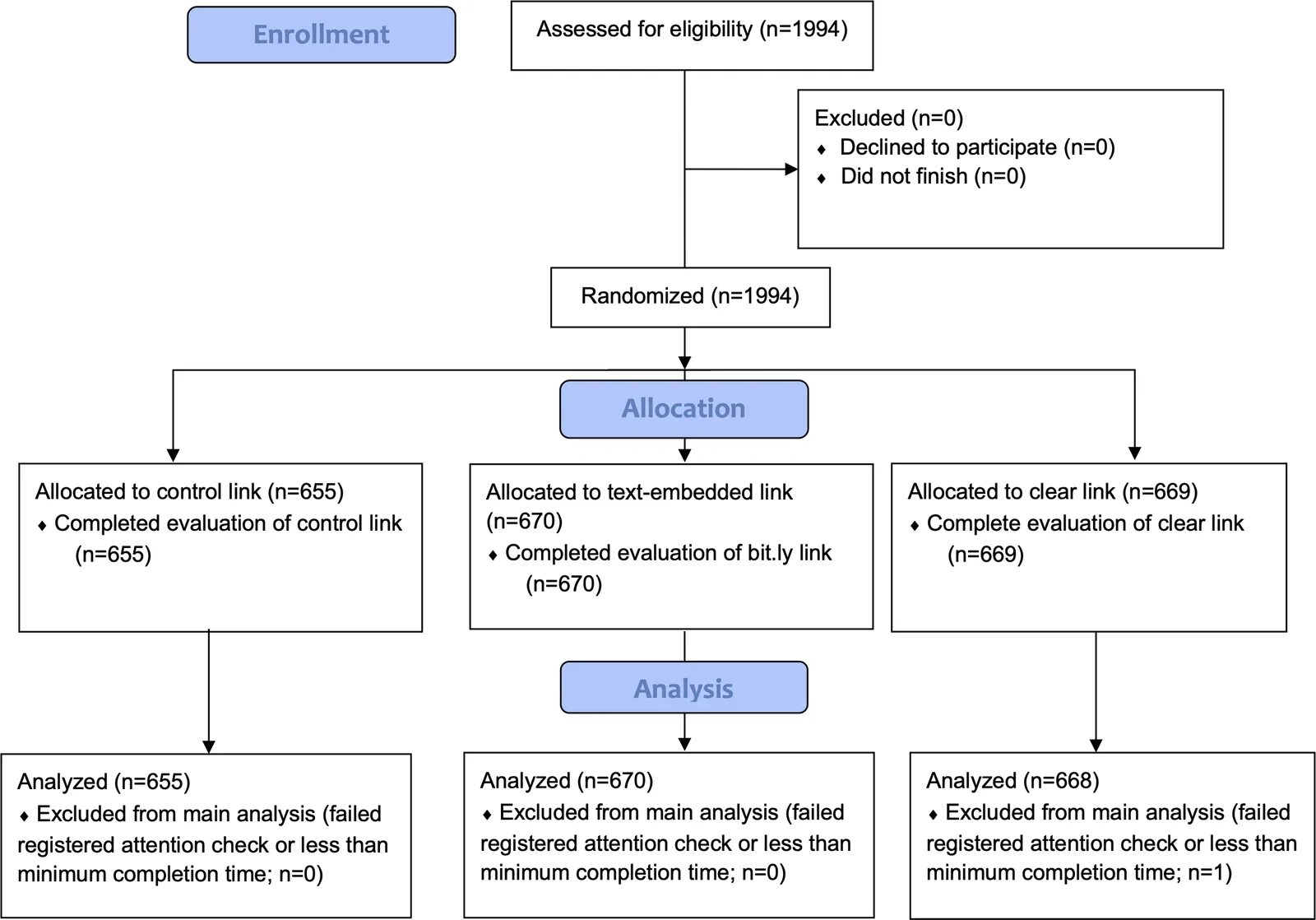
Participant flow chart for experiment 3. Exclusion decisions followed preregistered criteria: <5 minutes completion time or incorrectly answered 2 attention checks.

**Figure 9.**
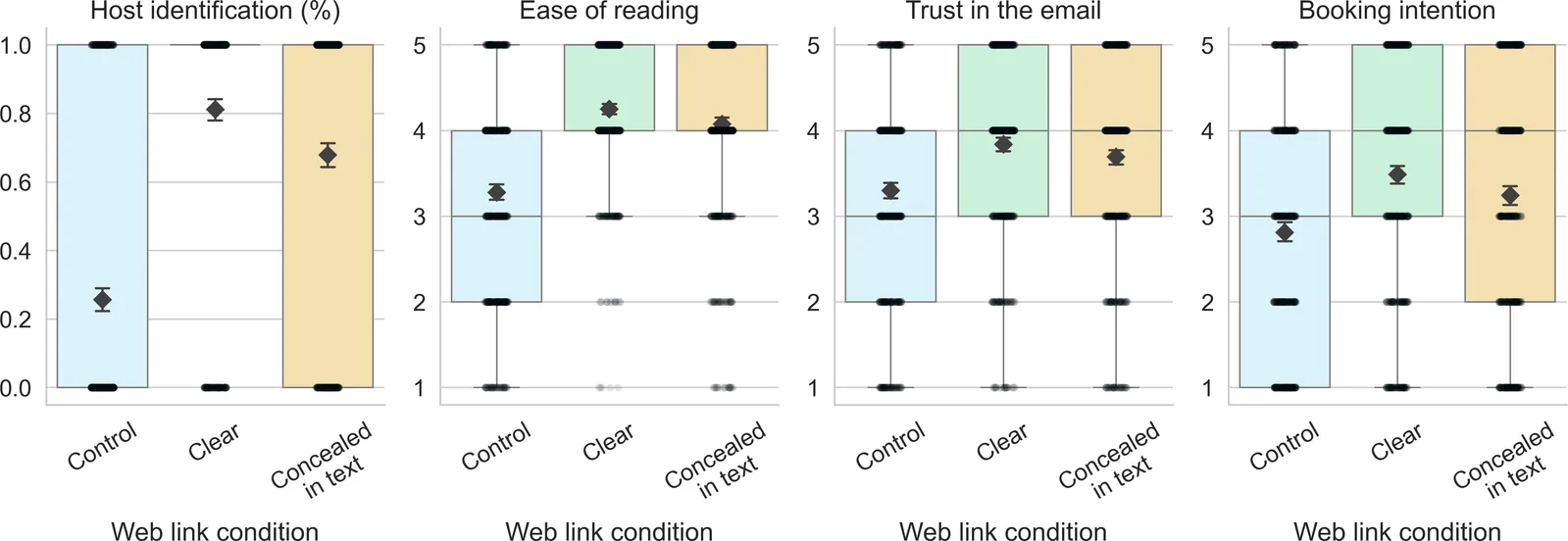
Relative to the control link (in blue), the clear web link (in green) and the text-embedded web link (in orange) helped participants recognize the website host correctly, were perceived as easier to read, and had an overall positive effect on email trust perception and booking intention (experiment 3, N=1993 UK participants; n=1009, 51% female; n= 959, 48% ethnic minority; February-March 2022). The means are shown in black diamonds with error bars showing 95% CIs. The box represents the IQR, the error bars around the box show the minimal and maximal values without outliers. The light gray circles show data points with slight horizontal jitter, so that wider and denser clusters represent more frequent answers. The host identification panel (leftmost) shows the proportion of correct interpretations (0: 0% and 1: 100%). The control link was the National Health Service (NHS) original vaccination invitation link “accurx.thirdparty.nhs.uk/r/aafwaczmd5,” the clear link was “https://vaccine-booking.nhs.uk” and the concealed text-embedded web link was “book here.”

**Figure 10.**
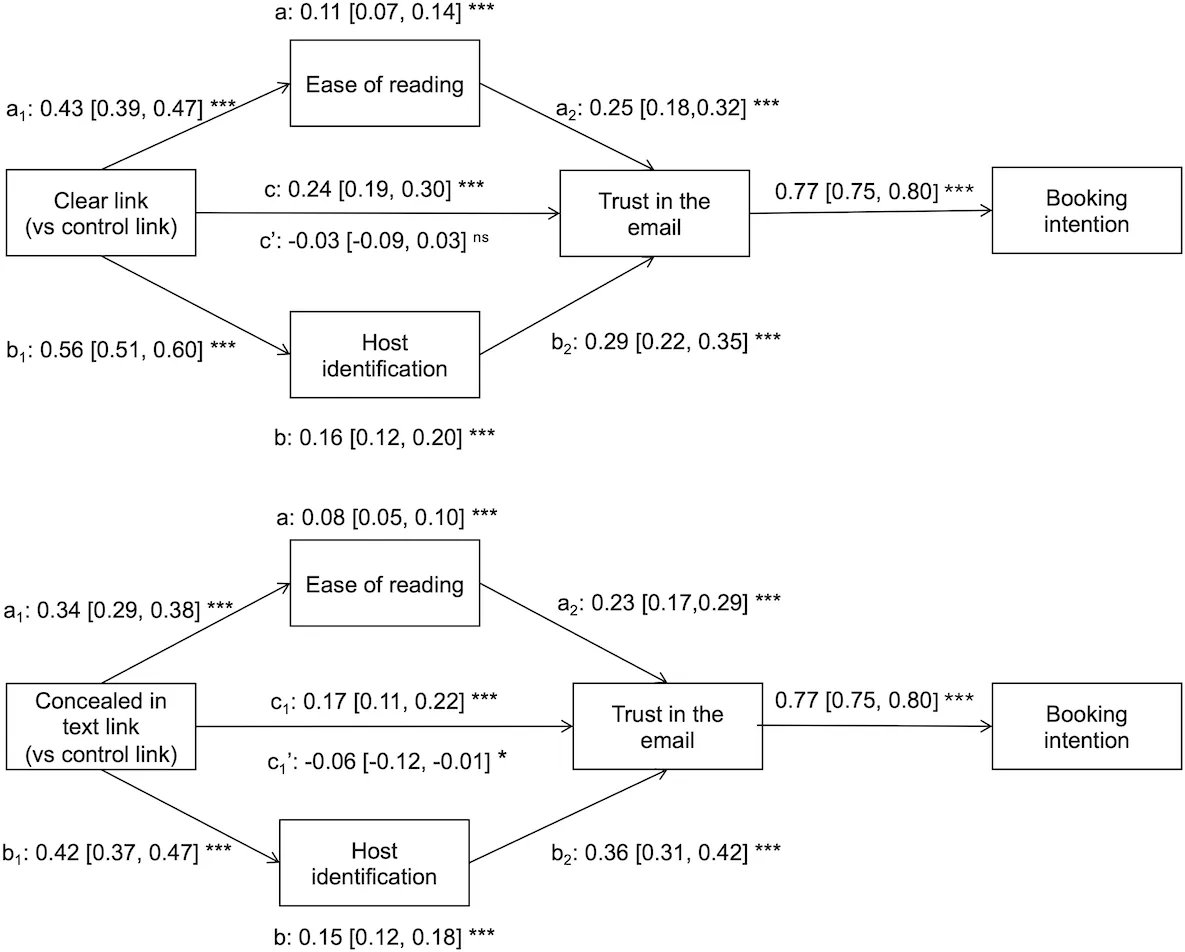
The clear web link and text-embedded web link improved trust and booking intention (vs control), and these effects were explained by the correct host identification and perceived ease of reading. The model shows β regression coefficients. (experiment 3, N=1.993 UK participants; n=1009, 51% female; n=959, 48% ethnic minority, February-March 2022). Indirect effects of the link: a: via ease of reading of the link, b: via correct host identification. Values are standardized, path analysis β coefficients with bias-corrected bootstrap 95% CIs from 5000 samples. ns: not significant. *P<.05, **P<.01, ***P<.001.

In experiment 3 (refer to Figure 8 for full CONSORT [39] participant flow chart), both the clear link and the text-embedded web link improved host identification (clear: 542/668, 81% correct; text-embedded: 455/670, 68%; control: 168/655, 26%, respectively), were perceived as easier to read, and increased trust and booking intention relative to the control condition, as illustrated in Figure 9. The improved host identification and perceived ease of reading mediated the positive effect of the clear and text-embedded web links on trust (Figure 10). The comparison of the 2 experimental conditions showed that the clear web link increased correct host identification and generated more trust in the email, along with higher booking intention than the text-embedded web link (illustrated descriptively in Figure 9 and analyses in Multimedia Appendix 1). The effect of each web link on trust, compared with the control link, was similar after adding gender, age, education, and ethnic minority status as covariates in a regression analysis (Multimedia Appendix 1), in this larger, more ethnically diverse sample (959/1993, 48% minority groups).

We tested the effect of the web links on ethnic minority participants separately and found consistent effects. The clear web link and the text-embedded web link increased correct host identification and ease of reading, which increased trust and booking intention (Multimedia Appendix 1). The clear link was more effective for ethnic minority participants. Post hoc ancillary analyses, in response to reviewer request, examined link effect interactions with age, education, gender, and ethnic minority status. These post hoc analyses showed that the improved links were increasingly effective as participant age increased (Multimedia Appendix 1). Improved trust perception was lower for the youngest participants in the sample, especially those who were women, participants from an ethnic minority group, or participants who did not have a tertiary (university) education.

The results of experiment 3 confirm the pivotal role of web links in vaccination invitation emails. We found that the design of the link shaped participants’ trust in the email and their intention to book their vaccination appointment via the web link. The study also found that the improvements to the control web link were effective for participants from ethnic minority groups, who tend to report lower levels of trust in health institutions and lower vaccination engagement [41-43].

## Discussion

### Principal Findings

We conducted 3 experiments to identify the characteristics of web links that enhance trust and engagement with realistic hypothetical digital vaccination invitations. In each experiment, and in support of our hypotheses, we found that easy-to-read (vs difficult) web links with clear (vs ambiguous) host organizations increased trust in vaccination invitation emails and vaccination booking intention. We also demonstrated that the control condition – a deactivated web link that had been used during the COVID-19 vaccination campaign – was poorly trusted by participants and led to lower intentions to book a vaccination appointment using the link.

This work complements research on how best to frame information about vaccine effectiveness [2,3,45,46], bringing attention to an element of digital communications that, to our knowledge, has been overlooked by behavioral research, that is, embedded web links [24]. By shifting analytical focus from message content to web link design features, this study extends existing health communication research and identifies that how web links are presented to recipients is an underexamined yet consequential trust cue in digital health contexts.

The COVID-19 pandemic required people nationwide to change their behaviors to protect themselves and their communities [47]. Timely vaccination uptake is facilitated by using digital invitation messages and reminders [5,6,48]. However, people are understandably skeptical when they receive a text message inviting them to click on a link. Scammers exploited people’s eagerness to be vaccinated and sent fake vaccination invitations, aiming to steal private information [8,15]. Our work on web links complements research focused on developing trustworthy digital (health) communication and on strategies that make people more likely to fall for phishing attempts (eg, time pressure, the threat of losing out on an opportunity) [22,24].

Consistent with information scent theory [21] and previous research on web links [24], we found that the features of web links shape their use. We found that difficult-to-read links harmed people’s perception of the message in which the web link appears, making the vaccination invitation emails look untrustworthy and reducing recipients’ willingness to use the link. Our control link, which was the web link used by the NHS in 2021 and 2022 during the COVID-19 vaccine rollout, was not easy to read and did not facilitate host identification. While the NHS link (“accurx.thirdparty.nhs.uk/r/aafwaczmd5”) did mention the name of the host, the host information was lost to recipients because this crucial information was crowded between a reference to a third party (“accurx.thirdparty”) and a string of letters and numbers (“r/aafwaczmd5”). The overall link is ambiguous, disfluent, and seemingly untrustworthy, making it unlikely to trigger user engagement. Despite the link being an actual NHS link that people in the United Kingdom had already received, fewer than half of the participants in our experiments (total n=3183) believed that clicking on the link would direct them to an NHS webpage. This suggests that the link undermined the participants’ trust in the email despite the presence of other cues that would make the email appear legitimate.

Our evidence showed that a web link with a clear reference to the NHS as the website host helped more recipients identify the host organization, which improved participants’ trust and user engagement with the web link when compared with the disfluent control link. When participants could correctly identify the link led to an NHS webpage, they trusted the email more and were more likely to use the link. A condition for the host identification to increase trust might be that the host is known and trusted—like the NHS in our study [49]; the same results may not extend to organizations that are perceived as less trustworthy, like pharmaceutical companies that manufacture the vaccines [50]. We would expect, for instance, a link that makes it easy to identify that it is hosted by a distrusted organization would have a negative effect on people’s trust in the email and their willingness to use the link. While we did not test the role of institutional trust, we replicated the benefits of host identification in a diverse sample (experiment 3), including ethnic minority groups who tend to trust health institutions less than the white majority group (eg, study by Hussain et al [41]), showing that the positive effect of identifying the NHS as the website host holds even for these segments of the population. Further research should test whether facilitating the identification of the host using a clear web link might show null effects for unknown or lesser-known hosts or even backfire for hosts with a negative reputation.

The extent to which people find text, like web links, easy to read and understand—known as fluency—is a meta-cognitive perception: a higher-level perception within cognitive processing [27]. When the NHS sent a vaccination invitation, the fluency of the web link had a role over and above whether participants recognized the website host. Easy-to-read links increased trust in the email and user engagement with the link itself, adding evidence of the effect of fluency on people’s judgments and decisions (eg, Fastinorbine [28-31]).

Should changing web links not be possible, we also found that concealing the links in text increases fluency perception, trust, and user engagement with invitation emails. Concealing links within embedded text also reduces the information people can get from links—specifically, not being able to draw from them who the host is and what the website’s function is. However, we found that concealing the link is not an issue in the context of an email that clearly features a host (eg, the email is sent from the NHS or includes the NHS logo). We found that, relative to our control link, “hiding” links in embedded text helped participants identify the website host, improved trust in the message, and increased the intention to use the link. Hiding links in a shortened URL (eg, “https://bit.ly/3GtTL0c”), on the other hand, did not improve trust and booking intention. It facilitated host identification, but they felt less fluent, despite being more concise (one operationalization of fluency [31]). Shortened links are senseless strings of numbers and mixed-case letters, making them difficult to read. Our evidence showed that hiding web links within text rather than using a shortened URL elicited greater trust. However, our third experiment showed that the links embedded in text were perceived as less trustworthy than a clear link, possibly because of the lack of transparency in the URL—participants could not identify where the link would lead and had to guess based on who sent the email.

In our studies, even hidden links—that did not feature the host organization—improved host identification and user engagement, but this was due to the presence of other cues in the email that could be used to identify the host or sender of the invitation. For example, in our experiments, the sender’s email address, the NHS branding in the email, and the tone of the message were consistent with real-world NHS digital communications in use at the time, which could have contributed to participant trust in the email. Hidden links may be particularly suboptimal in the context of SMS, as, unlike emails, additional cues, such as a verifiable email address or domain name, are not readily available to the reader, and they can be used to redirect users to an alternate (fraudulent) website [33].

Finally, exploratory ancillary analyses revealed that modifying the links had positive effects across ethnic groups, genders, and educational levels, but the benefits varied by age. In our studies, younger people were either equally suspicious (experiment 1‐2) or more suspicious (experiment 3) of the email. Our final experiment (experiment 3) showed that this was because younger participants remained equally suspicious of the email, even when it included the improved web link. While younger generations are “digital natives” (ie, exposed to digital technologies from birth [51]), they are not better at detecting scam (vs genuine) emails [52,53]. We speculate that the lack of effect of the link manipulation for younger groups might have been because of a lack of attention to that specific part of the email [54].

### Limitations

Our studies were conducted via hypothetical invitations to which participants reported hypothetical intentions, which may not truly reflect how people respond to a real-life email. Further research should replicate these results in real life (eg, split [A/B testing]) and broaden the scope to other digital health communications such as appointments for tests or health checks (eg, studies by van Zutphen et al [55] and Murphy et al [56]). Future work should include the examination of invitations sent via SMS text messages, as these messages often lack verifiable branding cues that could enhance their perceived trustworthiness [33].

It should be acknowledged that additional factors may shape people’s perceptions of digital health invitations, such as people’s preexisting level of trust in the health organization [57] or whether they are a regular internet user [5,6,48]. Furthermore, other aspects of web links—that we did not manipulate—are likely to be relevant to assess trustworthiness, such as recognized internet domains (eg, “.gov”) and website security (eg, “https”) [20], although understanding this requires some information technology knowledge. Further research on the psychological effects of web links should include controls for individual differences in internet use or personal knowledge or experience of internet scams. Decisions to book vaccination appointments are also shaped by influences beyond the invitation email itself, such as perception of vaccines [25], the experiences or advice of others [58], and the subjective vaccination norms within their social group [59].

An important ethical consideration is that while these findings can help optimize public health messaging, they may also be misused by cybercriminals in scams and phishing attempts. For example, cybercriminals could mimic the link characteristics shown to enhance trust—such as clear web links and text-embedded web links—to deceive recipients. This underscores the importance of pairing communication design improvements with strong public education around digital vigilance. Users should be regularly reminded to verify message sources, check for secure and official URLs, and report suspicious content. Public campaigns should aim not only to improve the effectiveness of health communications but also to raise awareness about the evolving tactics used by cybercriminals.

### Recommendations for Future Health Communications

In a context where real vaccination invitations must battle against scam messages to garner the recipient’s trust [11], our findings show that the design of web links can shape overall trust in the communication. This has direct real-world implications for health care organizations that rely on digital outreach, suggesting that seemingly minor design decisions—such as URL features that improve fluency and the identification of trustworthy hosts—can meaningfully affect public trust and engagement with health recommendations. While hidden links can be a way to sidestep fluency issues, they also carry risks because they lack transparency [33]. They can be easily used by cybercriminals to redirect users in phishing attacks, and hence, clear links might be a better option overall. Therefore, we recommend that future public health messaging, whether for pandemic response or as part of regular preventative medicine, health program, or disease management, consider the ease of reading and format of web links in addition to the message or email content and ensure that recipients can easily verify the sender of the email or text message to achieve optimal engagement in future health campaigns.

### Conclusion

Digital health communication designers face an uphill battle to secure users’ trust in a context where scams proliferate [4]. We extend digital communication research by investigating how web link design can reduce people’s justified suspicion in health messaging. Moving beyond the previous research focus on health message content (eg, study by Bushar et al [5]), we experimentally test the role of web link wording. We provide causal evidence that easier-to-read links that have an easy-to-identify host health institution increased trust and intention to use the link. Our work provides simple, practical design guidance to improve real-world engagement with digital health communications.

## Supplementary material

10.2196/85480 Multimedia Appendix 1 Other analyses performed, including subgroup analyses.

10.2196/85480 Checklist 1 CONSORT checklist.

## Abbreviations

CONSORT: Consolidated Standards of Reporting Trials
NHS: National Health Service
OSF: Open Science Framework
WHO: World Health Organization
