# Supplementary material for: Optimizing Web Links Used in Digital Vaccination Invitations to Raise Trust and Booking Intention: 3 Online Randomized Controlled Trials
Source: J Med Internet Res. 2026 Aug 25;28:e85480. doi: 10.2196/85480 (PMC13505598; doi:10.2196/85480)
Supplement: Supplementary file 1 [file jmir-v28-e85480-s001.docx]

# Multimedia Appendix

**Related to the following manuscript:**

Oakley, C. M., Sayer, H., Holford, D. L., Bruine de Bruin, W., Vallée-Tourangeau, G., Chadborn, T., Sirota, M., & Juanchich, M. (2026). Optimizing Weblinks Used in Digital Vaccination Invitations to Raise Trust and Booking Intention: 3 Online Randomized Controlled Trials. *Journal of Medical Internet Research*, *28*(e85480). https://doi.org/10.2196/85480

Contents

Multimedia Appendix	1

A.	Experiment 1. Detailed sample description	2

B.	Experiment 1. Departure from the preregistered plan	5

C.	Experiment 1. Results of preregistered analyses	6

D.	Experiment 1. Distribution of variables and zero order correlations between variables	7

E.	Experiment 1. Results with the participants who failed the study attention checks	8

F.	Experiment 1. Effect of the weblink on trust while controlling for gender, age, education and ethnicity	10

G.	Experiment 2. Detailed sample description	11

H.	Experiment 2. Comparison of the two concealed links	15

I.	Experiment 2. Results of preregistered analyses	16

J.	Experiment 2. Effect of the concealed links on trust while controlling for gender, age, education and ethnicity	17

K.	Experiment 3. Detailed sample description	19

L.	Experiment 3. Results of the preregistered analyses	22

M.	Experiment 3. Effect of the clear link and the text embedded link on trust and booking intention as a function of ethnic group (minority vs. majority).	23

N.	Experiment 3. Comparison of the text embedded link and the clear link	25

O.	Experiment 3. Effect of the text embedded link and the clear link on trust, while controlling for gender, age, education and ethnicity	26

P.	Exploratory moderation analyses. Does the effect of the link change as a function of participants’ gender, age, education and ethnicity?	28

## A. Experiment 1. Detailed sample description

**Table SM1**

Characteristics of participants in experiment 1 (N = 569 UK residents)

|  | *n* | % |
| --- | --- | --- |
| Age group (years) |  |  |
| 18-24 | 70 | 12% |
| 25-34 | 147 | 26% |
| 35-44 | 132 | 23% |
| 45-54 | 89 | 16% |
| 55-64 | 98 | 17% |
| 65-99 | 33 | 6% |
| Gender identity |  |  |
| Man | 283 | 50% |
| Woman | 276 | 49% |
| Non-binary | 8 | 1% |
| Other | 2 | 0.4% |
| Ethnic minority | 83 | 15% |
| Native English speaker = Yes | 521 | 92% |
| English proficiency in non-native |  |  |
| Beginner | 0 | 0% |
| Intermediate | 1 | 2% |
| Advanced | 23 | 48% |
| Expert | 24 | 50% |
| Level of education |  |  |
| No formal education | 1 | 0.2% |
| Primary | 1 | 0.2% |
| GCSE | 87 | 15% |
| A/AS Levels | 123 | 22% |
| Bachelor’s degree or equivalent | 229 | 40% |
| Master’s, Doctorate or equivalent | 122 | 21% |
| Other | 6 | 1% |
| Work Status |  |  |
| Working full time (30 or more hours per week) | 284 | 50% |
| Working part-time (1-30 hours a week) | 88 | 16% |
| Full-time student | 38 | 7% |
| Retired | 51 | 9% |
| Unemployed | 45 | 8% |
| Not working/homemaker | 47 | 8% |
| Other | 16 | 3% |
| Financial hardship during the COVID pandemic |  |  |
| Yes, most of the time | 24 | 4% |
| Yes, on a few/some occasions | 122 | 22% |
| No | 416 | 74% |
| Social media used on a regular basis |  |  |
| YouTube | 337 | 59% |
| Facebook | 353 | 62% |
| WhatsApp | 384 | 68% |
| Twitter | 189 | 33% |
| TikTok | 95 | 17% |
| Snapchat | 80 | 14% |
| Instagram | 263 | 46% |
| Reddit | 118 | 21% |
| Another platform | 6 | 1% |
| None of the above | 21 | 4% |
| Political party |  |  |
| Conservative | 116 | 20% |
| Labour | 179 | 32% |
| Liberal Democrat | 43 | 8% |
| Scottish National Party (SNP) | 17 | 3% |
| Plaid Cymru | 2 | 0.4% |
| Brexit Party/Independence party | 14 | 3% |
| Green Party | 73 | 13% |
| Other | 35 | 6% |
| Prefer not to say | 90 | 16% |
| Overall household income (after tax) |  |  |
| <£ 25,000 per year | 182 | 34% |
| £25,000 - £49,999 per year | 210 | 39% |
| £50,000 - £74,999 per year | 95 | 18% |
| £75,000 - £124.999 per year | 41 | 7% |
| > £125,000 per year | 11 | 2% |
| Urban/Rural |  |  |
| Urban | 223 | 41% |
| Suburban | 237 | 44% |
| Rural | 79 | 15% |
| Region |  |  |
| North-East | 26 | 5% |
| North-West | 60 | 11% |
| Yorkshire & Humber | 62 | 12% |
| East Midlands | 38 | 7% |
| West Midlands | 46 | 9% |
| East of England | 42 | 8% |
| London | 75 | 14% |
| Scotland | 41 | 8% |
| Northern Ireland | 17 | 3% |
| Religion |  |  |
| No religion | 305 | 54% |
| Christian | 137 | 24% |
| Protestant | 13 | 2% |
| Roman Catholic | 28 | 5% |
| Muslim | 14 | 3% |
| Buddhist | 14 | 3% |
| Sikh | 2 | 0.4% |
| Hindu | 7 | 1% |
| Jewish | 2 | 0.4% |
| Agnostic | 46 | 8% |
| Other | 11 | 2% |

## B. Experiment 1. Departure from the preregistered plan

In our preregistration we planned to test the hypotheses in both a UK and US version of the study. In the UK, designing materials for the experiment was relatively simple. All communications about the COVID-19 pandemic in general, and subsequent vaccination, were centralized. The NHS vaccination services managed the vaccination rollout and associated processes. It was therefore possible to design materials for the study based on the style of communications sent out by the NHS or other governmental bodies. In the US, vaccination is decentralized with no single organization responsible for vaccination of the population. Most often, US vaccinations were sent by local pharmacies, therefore no single organization would be recognized by US participants. Therefore, in parallel, we replicated the UK study materials in a US sample, but instead of the NHS featuring on the emails, our UK and US-based research team chose to use an unknown (fictitious) pharmacy as the host.

The results of our preregistered analysis of the UK and US versions of the study, show notable differences. These differences are likely due to the hypothetical nature of the host organization tested in the US vs. the existence of the well-known and trusted host institution tested in the UK. We therefore report the results separately. In the main manuscript and here, we focus on the results of testing the link manipulations in the UK sample using the NHS as the sender of the emails. The results of Experiment 1 in the US sample, using a fictitious organization, are reported in Supplementary Materials B, available via the OSF link: https://osf.io/7ze84/?view_only=553dc8477c58401bbee59d989e5a634a.

## C. Experiment 1. Results of preregistered analyses

In the data reported in the main manuscript, where we focus on UK respondents receiving an email from the NHS, our preregistered analyses were to test the effect of the link on the identification of the website host using a chi-square test and the effect of the link on perceived ease of reading, trust, and booking intention using variance analyses. For the manuscript, we reported regression analyses instead to keep the results more concise and consistent across the three studies. The preregistered analysis results are consistent with the regression analysis findings and are reported below.

The clear link showed improved performance across all the indicators: participants’ ability to correctly identify the host of the URL link as the NHS, judging the link as easier to read, perceiving the email as more trustworthy, and were more likely to use it to book their appointment, χ2 (2, N = 569) = 197.584, P < .001. There was a positive effect of the link manipulation on trusting the email, with those who saw the clear weblink having more trust in the email than those who saw the control link, *F*(1,567) = 41.88, P < .001, η2 = .07. This was also the case for perceived ease of reading, where participants that saw the clear weblink, finding it easier to read compared to the control weblink, *F*(1,567) = 373.191, P < .001, η2 = .40. There was also a significant effect of the link manipulation on booking intention, with participants reporting that they would be more likely to click on the clear weblink to book their appointment, compared to the control link, *F*(1,567) = 39.59, P < .001, η2 = .07.

## D. Experiment 1. Distribution of variables and zero order correlations between variables

Figure SM1

*Distribution of and zero order correlations between variables in experiment 1*

## E. Experiment 1. Results with the participants who failed the study attention checks

In Experiment 1, from the sample of 614 UK residents, 13 did not respond to these questions, and 32 were excluded from our analyses for failing an attention check based on preregistered criteria. Here, we report the mediation analyses on the full sample, including those who failed the attention checks.

Differences from the results reported in the manuscript are highlighted in yellow in Figure SM2. The main deviation from the results reported in the main manuscript is that the fluency mediator of the effect of the clear (vs. control) weblink on trust is statistically significant. The a-path (mediation of the effect of the clear link on trust) had a *P* value of .067 after excluding data from participants who failed one or two attention checks, whereas with those data included, the *P* value is .048.

Figure SM2

*Mediation models testing showing the effect of the clear (vs. control) link on perceived ease of reading and host identification, trust and booking intention with (upper panel) and without (lower panel) data exclusions.*

Note: *** *P* < 0.001, ns: not significant. Indirect effects of the link: a: via perceived ease of reading of the link, b: via correct host identification. Values are standardized, path analysis β coefficients with 95% confidence intervals, with bias-corrected bootstrap 95% confidence intervals from 5000 samples. The yellow highlights indicate where the results without data exclusion (for speeding) differ from the results with data exclusion.

## F. Experiment 1. Effect of the weblink on trust while controlling for gender, age, education and ethnicity

We tested the robustness of the effect of the weblink manipulation on participants’ perceived trust in the email while controlling for sociodemographic characteristics. conducting regression analyses. As shown in Table SM2, after controlling for gender, age, education and belonging to an ethnic minority group, we found that the clear link still benefitted recipients who trusted the email in which it appeared more, relative to the control NHS link.

Table SM2

*The clear weblink improved trust perception even after we controlled for gender, age, education and whether participants belonged to an ethnic minority group.*

| Link manipulation: Clear link (vs. control) | B CI[] | t | Beta |
| --- | --- | --- | --- |
| Constant | 2.88 [2.37, 3.40] | 10.94*** |  |
| Link Manipulation | 0.56 [0.38, 0.73] | 6.31*** | 0.26 |
| Gender (Men0/Women1) | 0.14 [-0.03, 0.32] | 1.62 | 0.07 |
| Age | <0.01 [-0.00, 0.01] | 1.41 | 0.06 |
| Education | 0.06 [-0.03, 0.14] | 1.30 | 0.05 |
| Ethnic minority (vs. majority) | 0.17 [-0.08, 0.42] | 1.32 | 0.06 |
| Model | *F*(5, 558) = 9.88, *P* < .001, R = 0.29 | | |

Note: B: unstandardized coefficient. *** *P* < .001 * *P* < .05

## G. Experiment 2. Detailed sample description

**Table SM3.**

Characteristics of participants in Experiment 2 (N = 596 UK residents)

|  | *n* | % |
| --- | --- | --- |
| Age group (years) |  |  |
| 18-24 | 61 | 10% |
| 25-34 | 145 | 24% |
| 35-44 | 121 | 20% |
| 45-54 | 78 | 13% |
| 55-64 | 97 | 16% |
| 65-99 | 94 | 16% |
| Gender Identity |  |  |
| Man | 292 | 49% |
| Woman | 301 | 51% |
| Non-binary | 3 | 1% |
| Other | 0 | 0% |
| Ethnic minority | 82 | 14% |
| Native English speaker = Yes | 565 | 95% |
| English proficiency in non-native |  |  |
| Beginner | 0 | 0% |
| Intermediate | 6 | 19% |
| Advanced | 9 | 29% |
| Expert | 16 | 52% |
| Level of education |  |  |
| No formal education | 5 | 1% |
| Primary | 7 | 1% |
| GCSE | 177 | 30% |
| A/AS Levels | 151 | 25% |
| Bachelor’s degree of equivalent | 176 | 30% |
| Master’s, Doctorate or equivalent | 68 | 11% |
| Other | 12 | 2% |
| Work Status |  |  |
| Working full time (30 or more hours per week) | 287 | 48% |
| Working part-time (1-30 hours a week) | 85 | 14% |
| Full-time student | 17 | 3% |
| Retired | 93 | 16% |
| Unemployed | 53 | 9% |
| Not working/homemaker | 49 | 8% |
| Other | 12 | 2% |
| Financial hardship during covid |  |  |
| Yes, most of the time | 70 | 12% |
| Yes, on a few/some occasions | 208 | 35% |
| No | 312 | 52% |
| Social media used on a regular basis |  |  |
| YouTube | 318 | 53% |
| Facebook | 434 | 73% |
| WhatsApp | 381 | 64% |
| Twitter | 183 | 31% |
| TikTok | 146 | 25% |
| Snapchat | 130 | 22% |
| Instagram | 299 | 50% |
| Reddit | 36 | 6% |
| Another platform | 8 | 1% |
| None of the above | 48 | 8% |
| Political party |  |  |
| Conservative | 161 | 27% |
| Labour | 197 | 33% |
| Liberal Democrat | 64 | 11% |
| Scottish National Party (SNP) | 21 | 4% |
| Plaid Cymru | 9 | 2% |
| Reform UK (formerly Brexit Party) | 14 | 3% |
| Green Party | 25 | 4% |
| Other | 27 | 5% |
| Prefer not to say | 69 | 12% |
| Overall household income (after tax) |  |  |
| <£25,000 per year | 209 | 35% |
| £25,000 - £49,999 per year | 219 | 37% |
| £50,000 - £74,999 per year | 74 | 12% |
| £75,000 - £124.999 per year | 35 | 6% |
| £125,000 – 149,999 per year | 5 | 1% |
| £150,000 or more per year | 19 | 3% |
| Prefer not to say | 35 | 6% |
| Urban/Rural |  |  |
| City | 237 | 47% |
| Town | 250 | 42% |
| Rural | 109 | 18% |
| Region (UK) |  |  |
| North East | 34 | 6% |
| North West | 54 | 9% |
| Yorkshire & Humber | 48 | 8% |
| East Midlands | 46 | 8% |
| West Midlands | 61 | 10% |
| East of England | 52 | 9% |
| London | 75 | 13% |
| Scotland | 38 | 6% |
| Northern Ireland | 16 | 3% |
| Religion |  |  |
| No religion | 224 | 38% |
| Christian | 198 | 33% |
| Protestant | 30 | 5% |
| Roman Catholic | 51 | 9% |
| Muslim | 36 | % |
| Buddhist | 1 | 0.2% |
| Sikh | 4 | 1% |
| Hindu | 10 | 2% |
| Jewish | 2 | 0.3% |
| Agnostic | 21 | 4% |
| Other | 19 | 3% |

## H. Experiment 2. Comparison of the two concealed links

Compared to the shortened link, the text-embedded weblink helped participants to identify the website host (68% v 39%) and was perceived easier to read. The text-embedded weblink also led to greater perceived trust in the email. The effect was mediated by improved identification of the NHS as the website host and the fluency of the link (Figure SM5).

Figure SM3

*Results of the path analysis for effect of the text-embedded weblink on trust perception compared to the shortened weblink, resulting in increased booking intention; the effect on trust perception was mediated by perceived ease of reading and correct identification of the URL host.*

Note: *** *P* < 0.001, ns: not significant. Indirect effects of the link: a: via perceived ease of reading of the link, b: via correct host identification. Values are standardized, path analysis β coefficients with 95% confidence intervals, with bias-corrected bootstrap 95% confidence intervals from 5000 samples.

## I. Experiment 2. Results of preregistered analyses

As planned in our preregistration, we conducted the analyses separately for the UK and the US samples because the hypothetical nature of the organization used in the US changed the effect of recognizing the host. The results for the US samples are reported in Supplementary Materials B, accessed via the OSF https://osf.io/7ze84/?view_only=553dc8477c58401bbee59d989e5a634a.

In the data reported in the main manuscript, where we focus on UK respondents receiving an email from the NHS, our chi square and ANOVA preregistered analyses show an effect of the link manipulation on the recognition of the host, on perceived ease of reading, and on trust and intention to use the link (consistent with those we report in the manuscript using a mediation analysis).

There was a significant effect of the link manipulation on participants’ ability to correctly identify the host of the URL link as the NHS, χ^2^ (6, *N* = 596) = 149.389, *P < .*001. Participants most frequently identified the host of the URL in the text-embedded weblink condition. Participants also correctly identified the host of the shortened weblink compared to the control link—but were also not sure of the host just as often. The link manipulation had a clear effect on fluency perception, *F*(2,567) = 373.191, *P* < .001, η^2^ = .40, trust in the email *F*(2,593) = 7.68, *P* = .001, η^2^ = .03, and intention to use the link, *F*(1,593) = 5.84, *P* = .003, η^2^ = .02.

## J. Experiment 2. Effect of the concealed links on trust while controlling for gender, age, education and ethnicity

We tested the robustness of the effect of the weblink manipulation on the participants’ perceived trust in the email while controlling for sociodemographic characteristics. conducting regression analyses. As shown in Table SM4, after controlling for gender, age, education and belonging to an ethnic minority group, we found that the text-embedded weblink still benefitted recipients, who trusted the email more than the email containing the control link. We did not test the effect of the shortened weblink with covariates as it did not statistically significantly improve trust.

**Table SM4.**

*The text-embedded weblink improved trust perception relative to the control link after we controlled for gender, age, education and whether participants belonged to an ethnic minority group (Experiment 2).*

| Link manipulation: Text-embedded link (vs. control) | B 95%CI[] | *t* | Beta |
| --- | --- | --- | --- |
| Constant | 2.51 [1.81, 3.21] | 7.04*** |  |
| Link Manipulation | 0.47 [0.23, 0.71] | 3.90*** | 0.20 |
| Gender (Men0/Women1) | -0.18 [-0.43, 0.07] | -1.43 | -0.07 |
| Age | 0.01 [0.00, 0.15] | 1.86 | 0.10 |
| Education | 0.12 [0.15, 0.23] | 2.23* | 0.14 |
| Ethnic minority (vs. majority) | -0.18 [-0.52, 0.16] | -1.04 | -0.05 |
| Model | *F*(5, 392) = 7.81, *P* < .001, *R* = 0.26 | | |
| Link manipulation: bit.ly shortened link (vs. control) |  |  |  |
| Constant | 2.47 [1.73, 3.20] | 6.57*** |  |
| Link Manipulation | 0.04 [-0.22, 0.29] | 0.27 | 0.13 |
| Gender (Men0/Women1) | -0.19 [-0.46, 0.08] | -1.37 | -0.07 |
| Age | 0.01 [-0.00, 0.14] | 1.35 | 0.07 |
| Education | 0.14 [0.03, 0.26] | 2.45** | 0.13 |
| Ethnic minority (vs. majority) | 0.02 [-0.37, 0.41] | -1.19 | 0.006 |
| Model | *F*(5, 398) = 2.11, *P* = .063, *R* = 0.16 | | |

Note: B: unstandardized coefficient. *** *P* < .001 * *P* < .05

## K. Experiment 3. Detailed sample description

Table SM5

Characteristics of participants in Experiment 3 (N = 1,993 UK residents)

|  | *n* | % |
| --- | --- | --- |
| Age group (years) |  |  |
| 18-24 | 232 | 12% |
| 25-34 | 379 | 19% |
| 35-44 | 353 | 18% |
| 45-54 | 388 | 20% |
| 55-64 | 322 | 16% |
| 65-99 | 318 | 16% |
| Gender identity |  |  |
| Man | 981 | 49% |
| Woman | 1,009 | 51% |
| Non-binary | 2 | 0.1% |
| Other | 1 | < 0.1% |
| Ethnicity minority | 959 | 48% |
| Native English speaker = Yes | 1,772 | 89% |
| English proficiency in non-native |  |  |
| Beginner | 0 | 0% |
| Intermediate | 50 | 23% |
| Advanced | 100 | 45% |
| Expert | 71 | 32% |
| Level of education |  |  |
| No formal education | 17 | 1% |
| UK Primary | 22 | 1% |
| UK GCSE | 484 | 24% |
| UK A/AS Levels diploma | 497 | 25% |
| Bachelor’s degree or equivalent | 659 | 33% |
| Master’s, Doctorate or equivalent | 284 | 14% |
| Other | 30 | 2% |
| Religion |  |  |
| No religion | 628 | 32% |
| Christian | 678 | 34% |
| Protestant | 74 | 4% |
| Roman Catholic | 148 | 7% |
| Muslim | 230 | 12% |
| Buddhist | 23 | 1% |
| Sikh | 25 | 1% |
| Hindu | 73 | 4% |
| Jewish | 7 | 0.4% |
| Agnostic | 56 | 3% |
| Other | 51 | 3% |
| Work Status |  |  |
| Working full time (30 or more hours per week) | 848 | 43% |
| Working part-time (1-30 hours a week) | 339 | 17% |
| Full-time student | 100 | 5% |
| Retired | 336 | 17% |
| Unemployed | 158 | 8% |
| Not working/homemaker | 169 | 9% |
| Other | 43 | 2% |
| Financial hardship during the pandemic |  |  |
| Yes, most of the time | 143 | 7% |
| Yes, on a few/some occasions | 631 | 32% |
| No | 1,183 | 59% |
| Social media used on a regular basis |  |  |
| YouTube | 1,151 | 58% |
| Facebook | 1,256 | 63% |
| WhatsApp | 1,263 | 63% |
| Twitter | 573 | 29% |
| TikTok | 441 | 22% |
| Snapchat | 379 | 19% |
| Instagram | 948 | 48% |
| Reddit | 148 | 7% |
| Another platform | 32 | 2% |
| None of the above | 161 | 8% |
| Political party |  |  |
| Conservative | 541 | 27% |
| Labour | 770 | 39% |
| Liberal Democrat | 150 | 8% |
| Scottish National Party (SNP) | 44 | 2% |
| Plaid Cymru | 6 | 0.3% |
| Reform Party (Brexit/Independence Party) | 50 | 3% |
| Green Party | 94 | 5% |
| Other | 94 | 5% |
| Prefer not to say | 244 | 12% |
| Overall household income (after tax) |  |  |
| <£ 25,000 per year | 639 | 32% |
| £25,000 - £49,999 per year | 744 | 37% |
| £50,000 - £74,999 per year | 276 | 14% |
| £75,000 - £124.999 per year | 136 | 7% |
| £125,000 – £149,999 per year | 21 | 1% |
| £150,000 or more per year | 22 | 1% |
| Prefer not to say | 155 | 8% |

## L. Experiment 3. Results of the preregistered analyses

As planned in our preregistration, we conducted the analyses separately for the UK and the US versions of the study. The results for the US version are reported in Supplementary Materials, available via the OSF link: https://osf.io/7ze84/files/osfstorage (Juanchich et al., 2026).

In the data reported in the main manuscript, where UK participants read a vaccination email from the NHS, our chi square and ANOVA preregistered analyses show an effect of the link manipulation on the recognition of the host, on perceived ease of reading and on trust and intention to use the link (consistent with those we report in the manuscript using a mediation analysis).

There was a significant effect of the link manipulation on participants’ ability to correctly identify the host of the URL link as the NHS, χ^2^ (6, *N* = 1993) = 633.38, *P < .*001, c = 0.49. The link manipulation had a clear effect on fluency, *F*(2,1992) = 169.19, *P <* .001, η^2^ = .15, trust in the email, *F*(2,1992) = 40.32, *P <* .001, η^2^ = .04 and likelihood to click through, *F*(2,1992) = 39.26, *P <* .001, η^2^ = .04.

## M. Experiment 3. Effect of the clear link and the text embedded link on trust and booking intention as a function of ethnic group (minority vs. majority).

Figure SM4

*Relative to the control link (in blue), the clear weblink (in green) and the link concealed via text embedding (in orange)* *helped participants recognize the website host correctly, were perceived as more fluent, and had an overall positive effect on email trust perception and booking intention (the upper panel shows results for ethnic minority groups [n = 1,034] and Panel B shows results for the ethnic majority group [n = 959]).*

Figure SM5

*Results of the path analysis testing the effect of the clear weblink on trust perception compared to the text-embedded weblink in the subsample of participants from ethnic minority groups (n = 959).*

Note: *** *P* < 0.001, ns: not significant. Indirect effects of the link: a: via perceived ease of reading of the link, b: via correct host identification. Values are standardized, path analysis β coefficients with 95% confidence intervals, with bias-corrected bootstrap 95% confidence intervals from 5000 samples. Any non-White British respondents were categorized as belonging to an ethnic minority group.

## N. Experiment 3. Comparison of the text embedded link and the clear link

Relative to the text-embedded weblink, the clear link helped participants to identify the website host (68% vs. 81%) and was judged more fluent. The clear link also led to greater perceived trust in the email. The effect was mediated by improved identification of the NHS as the website host and the fluency of the link (Figure SM6).

Figure SM6

*Results of the path analysis for effect of the clear weblink on trust perception compared to the Text-embedded weblink, resulting in increased booking intention; the effect on trust perception was mediated by subjective fluency and correct identification of the URL host.*

Note: *** *P* < 0.001, ns: not significant. Indirect effects of the link: a: via perceived ease of reading of the link, b: via correct host identification. Values are standardized, path analysis β coefficients with 95% confidence intervals, with bias-corrected bootstrap 95% confidence intervals from 5000 samples.

## O. Experiment 3. Effect of the text embedded link and the clear link on trust, while controlling for gender, age, education and ethnicity

We tested the robustness of the effect of the weblink manipulation on participants’ perceived trust in the email by controlling for sociodemographic characteristics and second, we tested whether those characteristics moderated the magnitude of the effect of the weblink.

As shown in Table SM6, after controlling for gender, age, education, and an ethnic minority status, we found that the clear link and the text-embedded hyperlink still benefitted recipients. Participants trusted emails that contained clear or text-embedded links more than those that included the control NHS link. The analyses also showed an effect of age and education. People who were older and more educated trusted the clear and text-embedded links more than the control link.

**Table SM6.**

*The first regression model showed that the clear link and the text-embedded weblink improved trust perception relative to the control link after we controlled for gender, age, education and whether participants belonged to an ethnic minority group.*

|  | Clear link (vs. control) | | | Text-embedded link (vs. control) | | |
| --- | --- | --- | --- | --- | --- | --- |
|  | B CI[] | t | Beta | B CI[] | t | Beta |
| **Model 1: Link manipulation and covariates** | | | | | | |
| Constant | 3.05 [2.76, 3.34] | 20.74*** |  | 2.87 [2.56, 3.18] | 18.28*** |  |
| Link Manipulation (ctr0/xp1) | 0.56 [0.44, 0.68] | 8.97*** | 0.24 | 0.40 [0.27, 0.53] | 6.10*** | 0.17 |
| **Covariates** |  |  |  |  |  |  |
| Gender (Men0/Women1) | -0.13 [-0.26, 0.01] | -1.84 | -0.06 | -0.01 [-0.16, 0.13] | -0.16 | -0.01 |
| Age | 0.01 [0.00, 0.01] | 2.02* | 0.07 | 0.01 [0.00, 0.01] | 3.07** | 0.10 |
| Education  (high school0/tertiary1) | 0.18 [0.05, 0.30] | 2.78** | 0.08 | 0.18 [0.05, 0.31] | 2.65*** | 0.08 |
| Ethnic minority(1/maj0) | -0.01 [-0.14, 0.16] | 0.13 | -0.004 | -0.01 [-0.17, 0.14] | -0.14 | -0.01 |
| **Overall model 1** | *F*(5, 1297) = 20.39, *P* < .001, *R* = 0.27 | | | F(5, 1295) = 11.31, *P* < .001, *R* = 0.21 | | |

Note: B: unstandardized coefficient. CI: 95% confidence interval. *** *P* < .001, ** *P* < .01, * *P* < .05. Education was coded as 1 - undergraduate and above (tertiary), 0 – high school and lower.

## P. Exploratory moderation analyses. Does the effect of the link change as a function of participants’ gender, age, education and ethnicity?

With a larger sample, Experiment 3 also gave the opportunity to test interaction effects between the link manipulation and participant characteristics, to explore how effective the clear link and the text-embedded links were perceived across gender and age groups (Models 2 and 3 respectively, presented in Table SM7), education level and ethnic minority status (Models 4 and 5, in Table SM8).

**Table SM7.**

*The regression model showed that, in addition to the link having a main effect, the effect of the Clear link depended on participants gender (model 2) and age (model 3). The effect of the text embedded link only depended on participants age (model 3).*

|  | Clear link (vs. control) | | | Text-embedded link (vs. control) | | |
| --- | --- | --- | --- | --- | --- | --- |
|  | B CI[] | t | Beta | B CI[] | t | Beta |
| **Model 2: Link manipulation, covariates and interaction: gender x link manipulation** | | | | | | |
| Constant | 3.00 [2.71, 3.29] | 20.13*** |  | 2.84 [2.53, 3.16] | 17.86*** |  |
| Link Manipulation | 0.68 [0.51, 0.85] | 7.83*** | 0.29 | 0.46 [0.27, 0.64] | 4.88*** | 0.19 |
| **Covariates** |  |  |  |  |  |  |
| Gender (Men0/Women1) | -0.00 [-0.18, 0.18] | -0.01 | 0.00 | 0.04 [-0.15. 0.24] | 0.44 | 0.02 |
| Age | 0.01 [0.00, 0.01] | 1.94 | 0.05 | 0.01 [0.00, 0.01] | 3.04** | 0.10 |
| Education  (high school0/tertiary1) | 0.18 [0.05, 0.30] | 2.74** | 0.06 | 0.18 [0.05, 0.31] | 2.63** | 0.07 |
| Ethnic minority (1/ maj0) | 0.01 [-0.14, 0.16] | 0.00 | -0.00 | -0.01 [-0.16, 0.15] | -0.07 | -0.00 |
| Gender x Link | -0.25 [-0.50, -0.01] | 2.56* | 0.21 | -0.11[-0.37, 0.15] | -0.84 | -0.04 |
| **Overall model 2** | *F*(6, 1296) = 17.73, *P* < .001, *R* = 0.28 | | | *F*(6, 1294) = 9.54, *P* < .001, *R* = 0.21 | | |
|  | |  |  |  | | |
| **Model 3: Link manipulation, covariates and interaction: age x link manipulation** | | | | | | |
| Constant | 3.29 [2.95, 3.63] | 19.00*** |  | 3.18 [2.82, 3.53] | 17.45*** |  |
| Link Manipulation | 0.12 [-0.24, 0.48] | 0.64 | 0.05 | -0.20 [-0.58, 0.18] | -1.04 | -0.08 |
| **Covariates** |  |  |  |  |  |  |
| Gender (Men0/Women1) | -0.12 [-0.26, 0.12] | -1.78 | -0.05 | -0.00 [-0.15, 0.14] | -0.06 | -0.00 |
| Age | 0.00 [-0.01, 0.01] | -0.11 | 0.08 | 0.00 [-0.01, 0.01] | 0.29 | 0.01 |
| Education  (high school0/tertiary1) | 0.17 [0.05, 0.30] | 2.71** | 0.08 | 0.18 [0.05, 0.31] | 2.63** | 0.07 |
| Ethnic minority (1/ maj0) | 0.01 [-0.14, 0.15] | 0.07 | -0.00 | -0.10 [-0.17, 0.15] | -0.12 | -0.00 |
| Age x Link | 0.01 [0.00, 0.02] | 2.56* | 0.21 | 0.01 [0.01, 0.02] | 3.31*** | 0.28 |
| **Overall model 3** | *F*(6, 1296) = 18.15, *P* < .001, *R* = 0.28 | | | *F*(6, 1294) = 11.32, *P* < .001, *R* = 0.22 | | |

Note: B: unstandardized coefficient. *** *P* < .001, ** *P* < .01, * *P* < .05. Education was coded as 1 - undergraduate and above (tertiary), 0 – high school and lower.

**Table SM8.**

*The interaction effect Education x Link tested in model 4 showed that education level did not contribute to the perception of the improved link compared to the control link. The Ethnic minority status * Weblink interaction (model 5) analyses indicated the improved links were more beneficial for ethnic majority participants; ethnic minority participants preferred the clear link compared to the control.*

|  | Link Manipulation: Clear link (vs. control) | | | Link Manipulation: Text-embedded link (vs. control) | | | | |
| --- | --- | --- | --- | --- | --- | --- | --- | --- |
|  | B CI[] | t | Beta | B CI[] | t | | Beta | |
| **Model 4: Link manipulation, covariates and interaction: education x link manipulation** | | | | | | | | |
| Constant | 3.04 [2.75, 3.34] | 20.19*** |  | 2.85 [2.54, 3.17] | | 17.85*** | |  |
| Link Manipulation | 0.58 [0.41, 0.75] | 6.74*** | 0.25 | 0.43 [0.25, 0.60] | | 4.73*** | | 0.18 |
| **Covariates** |  |  |  |  | |  | |  |
| Gender (Men0/Women1) | -0.13 [-0.26, 0.01] | -1.84 | -0.06 | -0.01 [-0.16, 0.13] | | -0.16 | | -0.01 |
| Age | 0.01 [0.00, 0.01] | 2.04* | 0.07 | 0.01 [0.00, 0.01] | | 3.07** | | 0.10 |
| Education  (high school0/tertiary1) | 0.20 [0.03, 0.38] | 2.25* | 0.09 | 0.21 [0.02, 0.40] | | 2.20* | | 0.09 |
| Ethnic minority1 (/ maj0) | 0.01 [-0.14, 0.16] | 0.13 | 0.00 | -0.01 [-0.16, 0.15] | | -0.06 | | -0.00 |
| Education x Link | -0.05 [-0.29, 0.20] | -0.39 | -0.02 | -0.06 [-0.31, 0.20] | | -0.43 | | -0.02 |
| Model | *F*(6, 1296) = 17.01, *P* < .001, *R* = 0.27 | | | *F*(6, 1294) = 9.45, *P* < .001, *R* = 0.21 | | | | |
|  |  |  |  |  | | | | |
| **Model 5: Link manipulation, covariates and interaction: ethnic minority x link manipulation** | | | | | | | | |
| Constant | 3.00 [2.70, 3.29] | 19.95*** |  | 2.80 [2.48, 3.11] | | 17.45*** | |  |
| Link Manipulation | 0.66 [0.50, 0.83] | 7.73*** | 0.29 | 0.54 [0.36, 0.71] | | 5.88*** | | 0.22 |
| **Covariates** |  |  |  |  | |  | |  |
| Gender (Men0/Women1) | -0.13 [-0.26, 0.01] | -1.84 | -0.06 | -0.01 [-0.15, 0.13] | | -0.14 | | -0.00 |
| Age | 0.01 [0.00, 0.01] | 2.04* | 0.07 | 0.01 [0.00, 0.01] | | 3.10** | | 0.11 |
| Education  (high school0/tertiary1) | 0.18 [0.05, 0.30] | 2.75** | 0.08 | 0.18 [0.05, 0.31] | | 2.64** | | 0.07 |
| Ethnic minority (1/maj0) | 0.12 [-0.07, 0.32] | 1.25 | 0.05 | 0.14 [-0.07, 0.34] | | 1.32 | | 0.06 |
| Ethnic minority x Link | -0.22 [-0.47, 0.02] | -1.78 | -0.08 | -0.30 [-0.54, -0.02] | | -2.14* | | -0.10 |
| Model | *F*(6, 1296) = 17.55, *P* < .001, *R* = 0.27 | | | *F*(6, 1294) = 10.21, *P* < .001, *R* = 0.21 | | | | |

Note: B: unstandardized coefficient. *** *P* < .001, ** *P* < .01, * *P* < .05. Education was coded as 1 - undergraduate and above (tertiary), 0 – high school and lower.

We observed significant gender, age, and ethnic minority group interactions with the link manipulation, but not for education. These interaction trends are represented in Figure SM10. Further examination of the sample revealed that female participants were, on average, younger, more likely to be from an ethnic minority group, and more highly educated than the male participants, suggesting confounders explained some of observed the interaction effects in Tables SM10 and SM11. From Figure SM7, in conjunction with the regression results, we believe that the Age x Link interaction is robust, meaning that the improved links improved trust more in participants aged from 30-40 years old and above. The link manipulations were less effective for younger participants, particularly younger women or those who were younger and less educated or younger and from an ethnic minority group.

**Figure SM7**

*Effect of the weblink on trust perception as a function of [Panel A] participants’ age and gender identity, [Panel B] participants’ age and ethnic minority vs. majority status and [Panel C] participants’ age and education level.*

*.*
